# Characterization of an In Vivo Neutralizing Anti-Vaccinia Virus D8 Single-Chain Fragment Variable (scFv) from a Human Anti-Vaccinia Virus-Specific Recombinant Library

**DOI:** 10.3390/vaccines9111308

**Published:** 2021-11-10

**Authors:** Ulrike S. Diesterbeck, Henrike P. Ahsendorf, André Frenzel, Ahmad Reza Sharifi, Thomas Schirrmann, Claus-Peter Czerny

**Affiliations:** 1 Division of Microbiology and Animal Hygiene, Department of Animal Sciences, University of Göttingen, Burckhardtweg 2, 37077 Göttingen, Germany; Henrike.ahsendorf@arcor.de; 2 Yumab GmbH, Science Campus Braunschweig Sued, Inhoffenstr. 7, 38124 Braunschweig, Germany; a.frenzel@yumab.com (A.F.); th.schirrmann@yumab.com (T.S.); 3 Center for Integrated Breeding Research, Department of Animal Sciences, University of Göttingen, Albrecht-Thaer-Weg 3, 37075 Göttingen, Germany; rsharif@gwdg.de

**Keywords:** scFv, vaccinia virus, recombinant antibody, D8

## Abstract

A panel of potent neutralizing antibodies are protective against orthopoxvirus (OPXV) infections. For the development of OPXV-specific recombinant human single-chain antibodies (scFvs), the IgG repertoire of four vaccinated donors was amplified from peripheral B-lymphocytes. The resulting library consisted of ≥4 × 10^8^ independent colonies. The immuno-screening against vaccinia virus (VACV) Elstree revealed a predominant selection of scFv clones specifically binding to the D8 protein. The scFv-1.2.2.H9 was engineered into larger human scFv-Fc-1.2.2.H9 and IgG1-1.2.2.H9 formats to improve the binding affinity and to add effector functions within the human immune response. Similar binding kinetics were calculated for scFv-1.2.2.H9 and scFv-Fc-1.2.2.H9 (1.61 nM and 7.685 nM, respectively), whereas, for IgG1-1.2.2.H9, the Michaelis-Menten kinetics revealed an increased affinity of 43.8 pM. None of the purified recombinant 1.2.2.H9 formats were able to neutralize VACV Elstree in vitro. After addition of 1% human complement, the neutralization of ≥50% of VACV Elstree was achieved with 0.0776 µM scFv-Fc-1.2.2.H9 and 0.01324 µM IgG1-1.2.2.H9, respectively. In an in vivo passive immunization NMRI mouse model, 100 µg purified scFv-1.2.2.H9 and the IgG1-1.2.2.H9 partially protected against the challenge with 4 LD_50_ VACV Munich 1, as 3/6 mice survived. In contrast, in the scFv-Fc-1.2.2.H9 group, only one mouse survived the challenge.

## 1. Introduction

Vaccinia virus (VACV) is the prototype of the genus *Orthopoxviruses* [1]. VACV was used as a heterologous vaccine against variola virus (VARV), the causative agent of smallpox. Cessation of vaccination after smallpox was declared eradicated in 1980 left an increasing susceptible population [2]. While VARV solely infects humans [2,3], zoonotic poxviruses, such as cowpox virus (CPXV) and monkeypox virus (MPXV), can also cause severe and sometimes fatal infections [4,5,6,7,8,9,10]. While vaccination is generally safe and effective for the prevention of smallpox, in well-documented cases of various adverse reactions in individuals, especially in immune-compromised humans, caused by licensed vaccines [11,12,13], vaccinia immune globulin (VIG) has been used for treatment [14,15,16,17]. Nevertheless, VIG prepared from human donors bears the risk of quality variances between batches [12] and, even though it is reduced, a risk for the transmission of pathogenic agents [18].

Two antigenic distinct forms of VACV are present [19]. The intracellular mature virus (MV) is the most abundant infectious form in *Orthopoxviruses* responsible for host-to-host transmission. Extracellular enveloped virus (EV) consists of an additional envelope and is thought to be important for dissemination within the host [19,20,21]. Targets for neutralizing and protective antibodies were identified for MV surface proteins A13, A17, A27, D8, H3, L1, A28, and EV surface proteins B5 and A33 [22,23,24,25,26,27,28,29]. One linear epitope, which is highly conserved among OPXVs, was mapped at the C-terminus of A13 (amino acid residues (aa) 59–69) [30]. Moreover, six linear epitopes were mapped on the A27 protein of OPXVs (epitope #4: aa region 9–14, epitope complex #1A-D: between aa 26 and 39, and epitope #5: aa region 68–71) [31]. Other studies discovered four epitope groups on the A27 protein of VACV (group I: aa residues 21–40; group II: discontinuous; group III: aa residues 81–100; and group 4: aa residues 91–110) [32]. Anti-B5 mAbs detected a conformational epitope (aa residues 22–130) [33], as well as two additional ones localized to the SCR1-SCR2 border, and in the stalk region [34]. Hitherto, five conformational antigenic sites were identified on the D8 protein [35], and neutralization of VACV was demonstrated only in the presence of complement [36,37].

The 32 kDa protein D8 is a type 1 membrane protein and plays a role in the adsorption of the poxvirus to the host cell [22,38]. The atomic structure revealed a carbonic anhydrase fold with a central positively charged crevice binding to chondroitin sulfate (CS) E on cell surfaces [22,36,38,39]. Sequence alignments of D8 orthologs suggest a structural conservation of this binding site [36]. A hexameric arrangement of D8 on the viral particle is proposed, mediated as a trimeric self-association of disulfide-bonded homodimers, which might increase the avidity of D8 to CS [39]. Cross-linking experiments suggest a spatial proximity to A21, a member of the entry-fusion complex [40]. VACV D8L knockout mutants exhibited reduced infectivity in a BALB/c mouse model [41], but replicated efficiently in cell culture [42]. Using an optimized D8 DNA vaccine approach in a BALB/c mouse model, high titers of neutralizing antibodies were induced, which were protective against a subsequent challenge with VACV WR [43]. The characterization of a panel of murine monoclonal antibodies revealed four distinct antigenic groups on the D8 surface. Most effective were antibodies blocking the chondroitin sulfate CS-E interaction sites at K41, R44, K108, and R220 [39]. In addition, D8 seems to possess a high- and a low-affinity binding region within the central crevice for CS-E and CS-A, respectively [44].

Phage display provides a robust technique to isolate monoclonal antigen-binding fragments, which can then be converted into other larger molecules or full-size antibodies [45]. Schmaljohn et al. [46] constructed a Fab phage display library from peripheral blood of one human donor. Here, we isolated the peripheral blood mononuclear cells of four donors immunized previously with Dryvax^®^ and amplified the genetic information of all IgG isotype heavy and light chains. We describe the selection, engineering, and full in vitro, as well as in vivo, characterization of an anti-D8 antibody derived from a human IgG-based phage display library.

## 2. Material and Methods

### 2.1. Immunization and Lymphocyte Preparation

Four human volunteers were immunized via scarification with Dryvax^®^ (Wyeth Laboratoires, Marietta, GA, USA) according to the manufacturer’s directions with a two-pronged needle. In detail, naïve volunteers received five punctures, and booster vaccination in previously immunized patients was performed with 15 punctures. Volunteer 1 was vaccinated five years ago, whereas Volunteer 2 was naive. Volunteers 3 and 4 were immunized more than 10 years ago. After 20 (Volunteer 1 and 2) or 28 (Volunteer 3 and 4) days postimmunization, approximately 500 mL peripheral blood was collected, respectively, and sera were tested in duplicates for the presence of circulating anti-vaccinia virus (VACV) IgG in an ELISA and Western blot. For the ELISA, three volunteers (Volunteer 5 to 7) were used as negative serum controls and to determine the unspecific background. Plates were coated either with 2 µg/mL VACV or BSA (as irrelevant protein). For the calculation of the antibody titer specific for VACV, the adsorption measured on VACV was corrected with the corresponding value determined on BSA. The cut-off value was calculated according to Frey et al. [47] for each respective dilution:

$$\mathrm{Cut}\;\mathrm{off}=\;\bar{X}+\mathrm{SD}\times f$$

with:

$\bar{X}$ = mean of independent control sera;

SD = standard deviation of independent control sera;

*f* = 3.372 for confidence level 95% [47]. 

Peripheral blood mononuclear cells were isolated using Ficoll-Paque PLUS density gradient (GE Bioscience, Freiburg, Germany). Total RNA was extracted from at least 10^7^ cells per volunteer with the RNeasy MiniKit (Qiagen, Hilden, Germany), followed by cDNA synthesis using oligohexamers (pdN_6_) (Invitrogen, Karlsruhe, Germany) according to the manufacturers’ instructions. For the Western blot, 5 μg gradient-purified VACV Elstree was separated on an SDS gel. Blots were incubated with human sera and detected with goat anti-human IgG HRP antibody.

### 2.2. Library Construction 

The amplification of the variable region of IgG-heavy and κ- and λ-light chains was performed, with degenerated primer set (BACK primers) binding to the first 23 bp of framework region (FR) 1 of the variable regions and primers binding to the first constant regions, either of human IgG1 to 4 or κ- and λ-light chains (FOR primers, Table S1). In parallel, the quality and integrity of the cDNA were monitored with a primer pair amplifying 788 bp of human glyceraldehyde 3-phosphate dehydrogenase (GAPDH, [48]). A 50 µL PCR reaction consisted of 1–2 µL cDNA, 1x PCR buffer (7.5 mM Tris HCl pH 9, 0.2 mM MgCl_2_, 5 mM KCl, 2 mM (NH4)_2_SO_4_; Biotools), 0.4 µM of one BACK primer, 0.4 µM of one FOR primer, 10 mM of each dNTP, and 2 U DNA polymerase (Biotools). Amplification was performed with 10 min denaturation at 95 °C, 25 cycles of 94 °C for 1 min, annealing at 58 °C for 1 min, elongation at 72 °C for 2 min, and final elongation for 10 min. The length and purity of the products were visualized by 1% agarose gel electrophoresis. The PCR products with a size of about 650 bp were purified with the DNA Clean & Concentrator-5-kit (Zymo Research Europe, Glasgow, Scotland). 

In the following second semi-nested PCR, the variable regions of heavy and light chains were amplified. The purified corresponding PCR products of each volunteer were pooled to equal amounts, of which 50 ng was used for amplification with Phusion polymerase and the 5xGC buffer (Finnzymes, Espoo, Finland). In the case of the variable region of the heavy chains, the BACK primer set used in the first PCR was combined with primers binding to FR4 (Table S2). Those primers were extended with 20 bp of an overlapping sequence coding for the (G_4_S)_3_ linker. The complementary part is coded by an overhang in the primers annealing to FR1 of the light chains. The reactions were incubated for 30 s at 98 °C, followed by 30 cycles at 98 °C for 30 s, 60 °C for 30 s, 72 °C for 30 s, and final elongation for 10 min. The variable regions with their overhangs were gel-purified (MinElute-Kit; Qiagen, Hilden, Germany). 

The formation of single-chain fragment variable (scFv) occurred with splicing by overlap extension. In the first step, 300 ng of mixed to equal amounts of heavy- and light-chain variable regions were joined in the absence of primers. In a second step, 5 µL of the connected products were reamplified with outer primers, including restriction sites for *Sfi*I (3′-GTC CTC GCA ACT GC*G GCC CAG CCG GCC* ATG GCC-HuVH BACK-5′) and *Not*I (3′-GAG TCA TTC TCG ACT T*GC GGC CGC*-HuJκ/λ FOR-5′). The scFvs were gel-extracted and cleaved, first by *Sfi*I (NEB, Frankfurt, Germany) and then by *Not*I (NEB, Frankfurt a. M., Germany). The fragments were ligated into the phagemid pCANTAB5E *N*-terminal of the internal E tag (GE Biosciences, Freiburg, Germany) and electroporated into *Escherichia coli*, TG1. Colonies were grown overnight, either on bioassay dishes (NUNC, Wiesbaden, Germany) or in 8 cm diameter petri dishes (Sarstedt, Germany) for titration on 2 × TYG-A (tryptone 16 g/L, yeast 10 g/L, NaCl 5 g/L, glucose 2%, ampicillin 100 µg/mL) agar (15 g/L). Colonies were scraped into 5 mL 2 × TYG-A-15% glycerol and stored at −80 °C. 

### 2.3. Cells and Viruses

The permanent monkey kidney cell line MA104 cultured in minimum essential medium (MEM) and supplemented with 7% fetal calf serum was used to propagate the VACV strains Elstree and Munich 1 (M1) (for references, see [49]). Infectivity titers were determined on 24-well plates (Nunc, Wiesbaden, Germany) and calculated as plaque-forming units (pfu/mL). For plaque reduction neutralization test, Vero cells (obtained from A. Mayr, Munich, Germany) cultured in MEM and supplemented with 5% fetal calf serum were used and maintained in the same way as MA104.

### 2.4. Gradient Purification of Vaccinia Viruses Elstree and Munich 1

Vaccinia viruses Elstree as well as Munich 1 (M1) were grown in MA104 cells for one day. Cells and virus in supernatant were harvested by pelleting at 13,700× *g* for 2 h. The pellets were resolved with 1 mM Tris-HCl, pH 9.0, and sonicated/freeze-thawed three times to lyse the cells. Cell debris and nuclei were removed by centrifugation (2200× *g*, 10 min). The supernatant was layered onto a 36% (*w*/*v*) sucrose cushion prepared in 1 mM Tris-HCl, pH 9.0. After ultra-centrifugation in an SW28 -rotor (Beckman Coulter GmbH, Krefeld, Germany) at maximum 112,700× *g* for 90 min, the pellet was resolved with 1 mM Tris-HCl, pH 9.0, and applied onto a 60%/40%/20% (*w*/*w* in 1 mM Tris-HCl, pH 9.0) sucrose discontinuous gradient and, again, ultra-centrifuged at maximum 40,018× *g* for 90 min in an SW40 rotor (Beckman Coulter GmbH, Krefeld, Germany). For each virus, the visible white viral band was collected and washed with 1 mM Tris-HCl, pH 9.0, to remove residual sucrose solution (maximum 111,160× *g* for 1 h). Finally, the pellets were resolved in 1 mM Tris-HCl, pH 9.0, and the protein concentrations were determined [50].

### 2.5. ScFv Selection Using Purified Vaccinia Virus Elstree

The scFv-phage library was panned using VACV Elstree in four rounds. Phages were rescued by infection of log phase *E. coli* TG1. The first phage rescue was performed from 6 × 10^9^ bacteria cells cultured in 1 l 2 × TYG-A. The cells were grown to an optical density at 600 nm (OD600 nm) of 0.4–0.5. M13K07ΔpIII (Hyperphage, Progen, Heidelberg, Germany) at a multiplicity of infection (MOI) of 30 were used to infect 150 mL of the bacterial suspension. The incubation was stationary at 37 °C for 30 min, followed by shaking with 250 rpm (Sartorius Certomat^®^ BS-1, Göttingen, Germany) under the same conditions. The infected cells were harvested by centrifugation (137,000× *g*/4 °C/20 min) and resuspended in 1 l 2 × TY-A-K (kanamycin 70 µg/mL). For further replication and phage production, the infected cells were incubated under gentle shaking at 30 °C overnight (Sartorius Certomat^®^ BS-1, Göttingen, Germany). Phages were precipitated two times by the addition of 1/5 volume 20% polyethylene glycol 8000/2.5 M sodium chloride (20% PEG/2.5 M NaCl). The phages were resuspended in 1 mL PBS/15% glycerol. Aggregates were removed by high-speed centrifugation (10,000× *g*/4 °C/1 min) and the supernatant stored at 4 °C overnight. For the successive selection rounds, 10^9^ bacteria cells were grown in 100 mL media. Twenty milliliters of the bacteria solution were infected with M13K07 MOI 30 (NEB, Frankfurt, Germany). Phage packaging was performed in 200 mL 2 × TY-A-K.

Unselected phages were used in a Western blotting assay on VACV Elstree gradient to determine the range of VACV proteins detected by the phage repertoire. The visualization occurred with HRP/anti-M13 monoclonal conjugate (GE Healthcare, Freiburg, Germany). 

For phage selection, one well of a 96-well MaxiSorb plate (Thermo Fisher Scientific, Langenselbold Site, Germany) was coated with 10 µg VACV Elstree diluted in 150 µL carbonate-bicarbonate buffer (pH 9.6) at 37 °C for 4 h and stored at 4 °C overnight. The well was washed three times using a Tecan washer (Tecan, Männedorf, Switzerland) for standardized washing procedure. The virus-coated and an additional empty well were blocked with 2% skimmed milk powder (SMP) and 10% FCS in PBS/0.1% Tween at 37 °C for 2 h. Approximately 1 to 5 × 10^12^ cfu phages diluted in 150 µL PBS/2% SMP/10% FCS/0.05% Tween were incubated in the pre-blocked well at room temperature for 1 h. The blocking solution of the coated well was replaced by the pre-blocked phages. Incubation occurred at room temperature for 2 h. Unbound phages were removed by washing 10 times in the first panning round, followed by a 15-times washing procedure using PBS/0.1% Tween in the successive rounds. Bound phages were eluted by enzymatic cleavage with 200 µL trypsin solution (10 µg/mL PBS) and an incubation period of 30 min at 37 °C. Ten microliters were added to 1 mL previously prepared log phase *E. coli* HB2151, while the remaining solution was used to infect 10 mL log phase *E. coli* TG1. The cells were streaked on 2 × TYG-A plates and incubated at 30 °C overnight. Phage input and output titers were calculated for each panning round as colony-forming units. Following each panning round, 176 *E. coli* HB2151 clones were precultured in 150 µL 2 × TYG-A in a Multiple Well Plate 96-Well (Sarstedt, Nümbrecht, Germany).

### 2.6. Screening of Randomly Selected scFv-Producing HB2151 Clones in Indirect ELISAs

In order to produce antibody fragments without pIII fusion, 0.5 µL of the *E. coli* HB2151 precultures were transferred into 100 µL 2 × TYG (0.1%)-A and incubated at 30 °C for 4 h. The expression was induced by the addition of IPTG to a final concentration of 2 mM dissolved in 50 µL 2 × TY-A by gentle shaking at 30 °C overnight (Sartorius Certomat^®^ BS-1, Göttingen, Germany). The cells were pelleted (137,000× *g*/4 °C/20 min) and the supernatants were applied in an ELISA for prescreening. Wells of two Maxisorb microtiter plates were coated either with 100 µL of 2 µg/mL VACV Elstree or BSA as a negative control. All subsequent steps were performed at room temperature. The plates were washed three times and blocked for 2 h with 300 µL/well PBS/2% SMP/0.1% Tween 20. The blocking solution was renewed with 50 µL. On each plate, 50 µL scFv supernatant was added. The monoclonal antibody (mAb) 5B4/2F2 binding to epitope 1A [31,35] of VACV A27 was used as a positive control, whereas the parapoxvirus orf-specific mAb 3C5 [51] was applied as a negative control for the coated virus. The wells were washed five times with PBS/0.1% Tween after 2 h of incubation. Bound scFvs were detected with an HRP-conjugated anti-E tag polyclonal antibody (Abcam, Cambridge, UK) diluted 1:5000, while the mAbs were detected with a polyclonal goat anti-mouse antibody (Dako, Hamburg, Germany) diluted 1:5000. After 10 times washing, 100 µL/well of 3, 3′, 5, 5′-tetramethylbenzidine (TMB) substrate was added and the covered plates were incubated for 20 min. The reaction was stopped with 50 µL/well of 1 M HCl and the absorbance was measured at 450 nm.

Plasmids of ELISA positive colonies were isolated from 5 mL media using the MiniPrep Kit (Qiagen, Hilden, Germany). The genes encoding the variable regions of the heavy (VH) and light (VL) chains were sequenced using vector-specific forward primers S1 (5′-CAA CGT GAA AAA ATT ATT ATT CGC-3′) and R1 (5′-CCA TGA TTA CGC CAA GCT TTG GAG CC-3′), and reverse primers S6 (5′-GTA AAT GAA TTT TCT GTA TGA GG-3′) and R2 (5′-CGA TCT AAA GTT TTG TCG TCT TTC C-3′). The sequences were analyzed with the DNAStar program (SeqMan Pro and MegAlign. Version 12.0. DNASTAR. Madison, WI, USA). The deduced amino acid sequences were used to classify the presumed family and germline origin by search of IMGT/V-QUEST [52,53].

### 2.7. Purification of Selected scFvs

Two monoclonal scFvs with high ELISA values were produced in one liter. The culture was centrifuged at 1500× *g* at 4 °C for 15 min. The supernatant was precipitated on ice on a tumbler with the same volume of saturated solution of ammonium sulfate for 1 h. Thereafter, the resuspension was centrifuged with 1500× *g* at 4 °C for 10 min. The pellet was resuspended in 5 mL 1 M Tris-HCl, pH 8.0. The periplasmatic fraction was collected by addition of 20 mL ice-cold 1 × TES (200 mM Tris-HCl, 500 mM ethylenediaminetetraacetic acid, 500 mM sucrose, pH 8.0) to the bacterial pellet. A total of 33 mL of 1/5 × TES was added. The suspension was incubated on ice while shaking for at least 30 min. MgSO_4_ was added to a final concentration of 5 mM. Centrifugation was conducted with 1500× *g* at 4 °C for 10 min. The supernatant contained the periplasmatic fraction of the scFv. 

Both filter-sterilized and pH 7.0 to 8.0 adjusted fractions were purified with an anti-E tag column (GE-Healthcare, Freiburg, Germany). The column was equilibrated with binding buffer (20 mM phosphate buffer, 0.005% NaN_3_, pH 7.0). The samples were applied and washed with binding buffer. After removing all unbound proteins, the scFv was eluted with 0.1 M glycine buffer, pH 3.0, into neutralization buffer (0.1 M Tris, 0.005% NaN_3_, pH 8.2) (10:1). The protein concentration was determined after dialyzing against PBS [50]. 

### 2.8. Competitive ELISA for Epitope Detection 

An inhibition ELISA with murine monoclonal antibodies should reveal the epitope recognized by specific binding of scFv. The associated epitopes of the mAbs on the VACV proteins are described in Table 1. Flat-bottom 96-well microtiter plates (Nunc MaxiSorp) were coated with 2 µg/mL VACV in carbonate/bicarbonate buffer (pH 9.6; 100 µL/well). After blocking with 2% skimmed milk and 10% fetal calf serum in PBS, purified scFv or mAb (Ab 1) adjusted to a starting concentration of 100 µg/mL was added in twofold serial dilutions (100 µL/well). The maximum extinction with 100 µg/mL was monitored in one well. Incubation was performed at room temperature for 2 h. After five washing steps with PBS, purified challenge scFv/mAb (Ab 2) was incubated under the same conditions. The concentrations of Ab 2 were determined empirically to ensure sufficient saturation of all free epitopes. The maximal extinction of Ab 2 was measured in an additional well coated with virus and not incubated with an inhibitory antibody. The detection of the Ab 2 occurred either with goat pAb to E tag (HRP) (1:2000) or goat anti-mouse IgG peroxidase conjugate developed in goat (1:2000) (whole molecule; Sigma Aldrich, Taufkirchen, Germany) at room temperature for 1 h. After washing five times with PBS, the developing solution (3, 3′, 5, 5′ tetramethylbenzidine; Abcam, Cambridge, UK) was added. The reaction was stopped by 1 *N* hydrochloric acid. The OD values were measured by a photometric plate reader (TECAN, Männedorf, Switzerland) at a wavelength of 450 nm. Reduction in the photometer extinction of detected challenge antibodies by competing antibodies was calculated as:

$$\%\left(\mathrm{inhibition}\right)\;=1-\frac{{\mathrm{OD}}_{450\mathrm{nm}}\left(\mathrm{Ab1}\mathrm{Ab2}\right)}{{\mathrm{OD}}_{450\mathrm{nm}}\left(\mathrm{Ab2}\right)}\times 100$$

An inhibition of at least 50% was regarded as a significant blocking effect.

### 2.9. Engineering of Specific Binding scFv to Human scFv-Fc and IgG1 Molecules

Specific binding scFv were converted into scFv-Fc and IgG formats. The addition of the second and third constant region of a human IgG1 enables studies on further effector mechanisms within the immune system. The binding affinities to VACV and neutralization abilities were compared. 

The pCANTAB5E, possessing the genetic information of the selected scFvs, and the vector pCMX2.5 were cleaved using the restriction enzymes *Nco*I and *Not*I. In total, 1 µg of each plasmid was first incubated at 37 °C for 1 h with *Nco*I and, after heat inactivation, incubated with *Not*I under the respective buffer conditions. The vector pCMX2.5 was dephosphorylated, while the scFv was gel-extracted. Ligation occurred at 16 °C overnight, followed by transformation into *E. coli* DH5α. Colonies were randomly selected. The successful ligation was confirmed by the sequencing of isolated plasmids. HEK293T cells (approximately 7.5 × 10^5^ cells) were seeded in growth medium (Dulbecco’s modified Eagle’s medium (DMEM) containing 5% (*v*/*v*) fetal calf serum (FCS) and 1% penicillin/streptomycin (PS)) into 6-well culture plates (Sarstedt, Germany) and were grown to reach a confluence of 75 to 80% for transfection after 24 h. A total of 20 µL of a 1 mg/mL polyethylenimine (PEI, linear, 25 kDa, Polysciences) solution was diluted in 125 µL DMEM. In parallel, 2 µg of purified pCMX2.5-scFv-Fc was diluted in 150 µL DMEM. PEI and DNA dilutions were combined and incubated for 15–30 min at RT to allow formation of PEI-DNA complexes. This suspension was dispersed over the cells and incubated overnight. The medium was changed to DMEM/4% FCS/1% PS for primary production of scFv-Fc into the culture supernatant. Immunoaffinity purification of the fragments occurred by Protein G column. 

For expression of IgG1, Fv fragments were cloned into IgG expression vectors pCSL3k (light chain) and pCSH1c (heavy chain). The VH and VL were PCR-amplified using primers TS_UDH9VH_*Bss*HII_f (5′-CACAGGCGCGCACTCCCAGGTGCAGCTGGTACA-3′) and TS_UDH9VH_*Nhe*I_r (5′-TGGTGCTAGCTGAGGAGACGGTGACCGT-3’) for the VH, and TS_UDH9VL_*Age*I_f (5’-AAGCACCGGTGAAATTGTGATGACGCAG- 3′) and TS_UDH9VL_*Bsi*WI_r (5′-CCACCGTACGTTTGATTTCCACCT-3′) for the VL. Cloning of VH and VL was carried out as described previously [54].

Monocistronic heavy- and light-chain vectors were co-transfected into HEK293-6E cells for transient expression [55]. In brief, heavy- and light-chain vectors were mixed with a molar ration of 1:1 and HEK293-6E cells at densities of 1.8–2 × 10^6^ cells/mL, transfected using PEI as described previously [56]. Forty-eight h after transfection, one volume of medium including 1% Tryptone N1 (TekniScience) was added for feeding. Supernatant was harvested 5 days after transfection. Immunoglobulins were purified by protein A affinity chromatography using the ProfiniaTM Affinity Chromatography Protein Purification System (Bio-Rad, Munich, Germany) according to the manufacturer’s description. 

### 2.10. SDS-PAGE and Western Blotting for the Detection of the Target Virus Protein

For Western blot analyses, 5 µg of gradient-purified VACV Elstree and 5 µg of the purified recombinant D8 protein were fractionated by vertical 12% sodium dodecyl sulfate (SDS)-polyacrylamid gel electrophoresis [57], and subsequently transferred to nitrocellulose membranes. Immunodetection was performed by standard techniques using 10 µg scFv, after a blocking step in PBS/2% SMP/10% FCS at room temperature for 2 h. Goat pAb to E tag (HRP) (1:500, Abcam, UK) and HRP color-developing reagent (Bio-Rad, Heidelberg, Germany) were used for visualization. For the verification of the virus gradient integrity an anti-MVA-polyclonal rabbit antibody (1:500) was used and visualized with polyclonal goat anti-rabbit IgG conjugated with HRP (Sigma-Aldrich, Taufkirchen, Germany). The protein sizes were estimated with a concurrent protein standard (Bio-Rad, München, Germany). The parapoxvirus orf D1701 was chosen as virus negative control and an anti-orf-polyclonal sheep Ab (1:500) was used for its verification.

### 2.11. Enzyme-Linked Immunosorbent Assay (ELISA) 

For quantification of the binding affinities, we modified the protocol according to Czerny and Mahnel [49]. The purified scFv, scFv-Fc, and IgG1 were titrated in triplicates in twofold serial dilutions, starting with 100 µM in an ELISA. Plates were coated with 2 µg/mL gradient-purified VACV Elstree. The background was determined with BSA and subtracted from the respective dilution measured on VACV Elstree. The affinity was calculated from the average adsorption of the triplicates using the Michaelis-Menten kinetics [58] and GraphPad Prism 6 for Mac (La Jolla, CA, USA).

The capture abilities of the scFv were tested in a sandwich ELISA. Plates were coated with 100 µL of 25 µg/mL scFv at 37 °C for 4 h, followed by 4 °C overnight. Blocking was performed after eight washings with PBS/0.1% Tween. The blocking solution was removed by washing eight times. The chosen virus samples, composed of cell culture supernatants, were titrated in twofold serial dilutions and incubated at 37 °C for 1 h. The tested viruses were reovirus (5 × 10^5.625^ CID_50_/mL), Newcastle disease virus (5 × 10^5.25^ CID_50_/mL), orf D1701 (10^4^ pfu/mL), enterocytopathogenic bovine orphan virus (5 × 10^5.25^ CID_50_/mL), bovine coronavirus V270 (5 × 10^5.25^ CID_50_/mL), and VACV Elstree (10^4^ pfu/mL) (virus strains obtained from A. Mayr and C.-P. Czerny, Munich, Germany). Negative controls were DMEM and the MA104 cell line in DMEM. After washing 10 times, the detection occurred with anti-MVA-polyclonal rabbit sera in a prior determined dilution of 1:5000. Binding of the detector was visualized with anti-rabbit IgG (whole molecule) peroxidase conjugate (Sigma-Aldrich, Taufkirchen, Germany) prediluted at 1:2000 and TMB substrate.

### 2.12. In Vitro Plaque Reduction Neutralization Test (PRNT)

To assess the neutralization abilities of scFv, scFv-Fc, and IgG1, a confluent monolayer of Vero cells was grown in 24-well culture plates. Antibodies were adjusted to 10 µM and titrated in triplicates in 2-fold serial dilution against approximately 50 pfu of VACV Elstree in MEM (PAN-BIOTECH GmbH, Aidenbach, Germany) and incubated at 37 °C for 1 h, with or without 1% human complement (Sigma Aldrich, Taufenkirchen, Germany). The virus-antibody (complement) mixture was then added to the cells and again incubated at 37 °C for 1 h under 5% CO_2_. The supernatant was removed and replaced with 0.5 mL of 2.5% FCS-0.5% methylcellulose in MEM, ensuring plaque formation within 48 h. Virus-positive control and an equal volume of MEM, with or without complement, was added to 50 pfu VACV Elstree. The neutralization positive control was anti-MVA-polyclonal rabbit sera. Fixation and staining of cells were carried out using 1.5% crystal violet in 8.5% ethanol/25% formaldehyde. Plaques were counted visually and a reduction in plaque number of ≥50% (half maximal inhibitory concentration, IC_50_) compared to the virus control was considered as significant virus neutralization.

### 2.13. In Vivo Neutralization

The mouse model was modified according to Czerny et al. [59]. To examine the neutralization abilities of scFv, scFv-Fc, and IgG1, seven groups containing six female NMRI mice aged 7 weeks were passively immunized intraperitoneally (i.p.) with 300 µL containing 100 µg of the corresponding antibody in PBS. Two groups of mice served as the negative control, receiving either PBS or an anti-equine-herpesvirus mAb. One group served as an antibody-positive control group, receiving a polyclonal anti-MVA antibody. Animals were challenged i.p. with 4 LD_50_ VACV Munich 1 24 h later. Mice were monitored for survival and signs of illness as the primary read-out. If ≥50% (IC_50_) of the mice were protected, a passive protection of the mice was demonstrated. Moreover, the body weight of each mouse was measured daily. Mice with a body weight loss of >30% were euthanized. The impact of weight was analyzed using the mixed procedure of SAS with the following model:

$$y_{\mathrm{ijklm}}=\mu +\alpha_{i}+\beta_{j}+\lambda_{k}+{\alpha \beta }_{\mathrm{ij}}+{\alpha \beta \lambda }_{\mathrm{ijk}}+b\left(G_{\mathrm{ijk}}\right)\;+\;\gamma_{l}+\varepsilon_{\mathrm{ijklm}}$$

where y_ijklm_ is the observation for weight, μ is the general mean, α_i_ is the effect of treatment, β_j_ is the fixed effect of survival ability, λ_k_ is the fixed effect of time, αβ_ij_ is the fixed effects of interactions between treatment and survival ability, αβλ_ijk_ is the fixed effects of interactions between treatment, survival ability and time, G_ijk_ is the starting individual weights, b is the regression coefficient, γ_l_ is the random effect of repeated measurement, and ε_ijklm_ represents the random error. 

Statistical analysis of the surviving mice was carried out by the Kaplan-Meier method using the LIFETEST procedure of SAS System 9.3 (SAS Institute Inc., Cary, NC, USA) and applying the following model:

$$\hat{S}\left(t\right)\;=\underset{j:t_{j}\leq t}{\prod }\left[1-\frac{d_{j}}{n_{j}}\right]for\;t_{1}\leq t\leq t_{k}$$

where *Ŝ*(*t*) is the survivor function and t is the lifetime of mice. For each *_j_: t_j_* ≥ *t*, let *t*_1_ < *t*_2_ < … < *t*_k_ represent the different event times. *n_j_* is the number of individuals at risk just prior to *t_i_*, and *d_j_* is the number of individuals that die at time *t_j_*. 

Tests of equality across strata were used to explore whether significant differences between different antibody treatment groups existed. Hazard rates were derived from the nonparametric survival function estimated with the Kaplan-Meier method.

At the end of the experiment (day 28 after challenge), heart, liver, spleen, lung, brain, and kidney were harvested. The viral load was quantitated by real-time PCR according to Czerny et al. [60]. Organ samples were weighed, and DNA was purified using QIAamp DNA Blood Mini Kit and QIAamp DNA Mini Kit (Qiagen, Hilden, Germany) according to the manufacturer’s instructions. Real-time PCR was performed on a LightCycler 480 (Roche, Mannheim, Germany) using the LightCycler 480 Probes Master Kit (Roche, Mannheim, Germany) to amplify the D8 fragment of VACV. The reaction volume contained 10 μL Light Cycler 480 Probes Master mix, 1 μL of each 5 pmol/μL D8L_forward: 5′-CATATTCATTGGGGAGAAACC-3′ and D8L_reverse: 5′-GCGATTGAAGACGTTAGACTAA-3′, 1 μL of 4 pmol/μL D8L_probe: 5′-TTCTGGATAGTGGTTGGTTTCGACTCA-3′, and 2 μL HPLC water, as well as 5 μL of the DNA template. The LightCycler was programed as follow: first, 10 min preincubation at 95 °C, then 40 cycles of 95 °C/30 s, 58 °C/45 s, and 72 °C/60 s, followed by a final cooling step at 40 °C for 10 min. Samples were amplified in duplicates. A log10 standard curve was established using DNA from gradient-purified VACV Elstree. Genome copies were calculated based on the standard curve and the sample weight. Following this, mean values per tissue of sacrificed and surviving animals were compared, as well as mean values over all animals per tissue.

## 3. Results

### 3.1. Immunization, Library Construction, and Characterization

**Figure 1:**
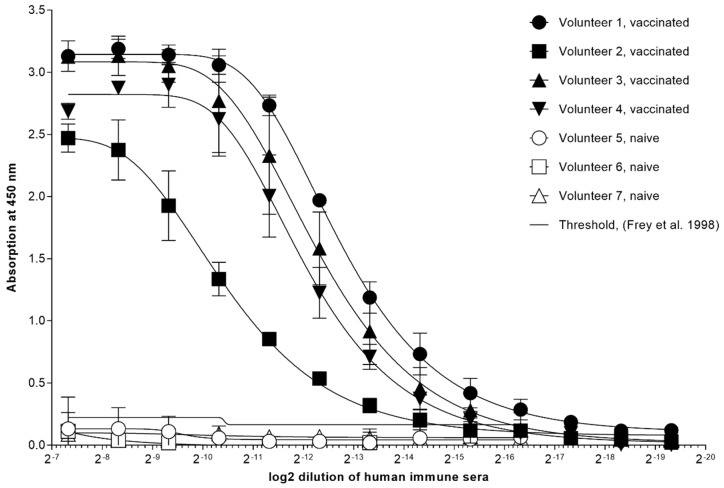
Circulating anti-VACV IgG in sera of four volunteers was measured by indirect ELISA. Blood samples were either taken 20 days (Volunteer 1 and 2) or 28 days (Volunteer 3 and 4) after immunization with Dryvax^®^ (Wyeth Laboratories, Inc., Marietta, GA, USA). Three volunteers (Volunteer 5 to 7) were not vaccinated. Sera were titrated in twofold serial dilutions.

Post-vaccination, volunteers developed pox-like lesions at the side of scarification. The size and degree of the reaction was about 0.5 × 0.7 cm in Volunteer 2 and only a reddish area of about 0.5 cm in diameter was visible for Volunteer 1. Lymphadenopathy of a few days was the major reaction. Volunteer 2 reported one day with fever. The titer of circulating anti-VACV IgG in the peripheral blood of four volunteers was measured by ELISA. The determined titers ranged between 1.024 × 10^4^ and 4.096 × 10^4^/mL serum. Volunteer 2, previously unvaccinated, revealed the lowest titer, whereas Volunteer 1, vaccinated several times, showed the highest anti-VACV titer (Figure 1). 

Neutralizing antibodies in the sera of the immunized individuals were determined through plaque reduction neutralization test using VACV Elstree. The sera showed neutralizing titers of 1.6 × 10^2^ to 3.2 × 10^2^/mL.

**Figure 2:**
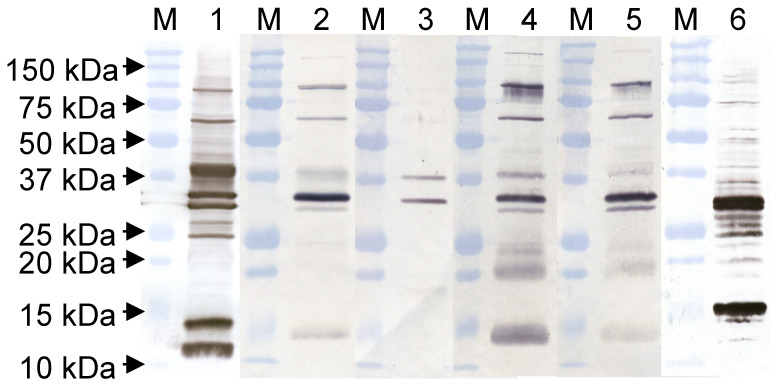
Western blot on 5 μg VACV Elstree gradient. Anti-MVA-polyclonal rabbit Ab (1) served as a positive control for VACV. Lanes 2 to 5 represent the proteins detectable by the volunteer sera (2 = Volunteer 1, 3 = Volunteer 2, 4 = Volunteer 3, 5 = Volunteer 4). With the polyclonal hyperphage preparation (6), a broad range of VACV proteins was verified. The used conjugates were anti-rabbit IgG (whole molecule) peroxidase conjugate developed in goat, anti-human IgG (Fc specific) peroxidase conjugate developed in goat, and anti-M13-HRP, respectively. M marks the protein ladder for each sample.

To construct the scFv library, RT-PCR was performed using total RNA of at least 10^7^ cells per volunteer. It was possible to amplify a specific product with every primer combination for every sample (Figures S1–S3). After pooling of the related 650 bp products and amplification of the variable regions with linker overhangs (Figure S4), the scFvs were joined by SOE-PCR (Figure S5). Subsequent to the ligation of scFv into pCANTAB5E, 40 transformations yielded in ≥4 × 10^8^ independent colonies. The unselected phage pool was tested in a Western blotting assay for binding to VACV proteins and revealed a broad range of proteins detected by the phages. The pattern of the detectable proteins showed similarities to the tested human sera of the four volunteers and the anti-MVA-polyclonal rabbit sera, but with different strengths of the bands (Figure 2).

### 3.2. Selection of Vaccinia-Virus-Specific scFv

**Figure 3:**
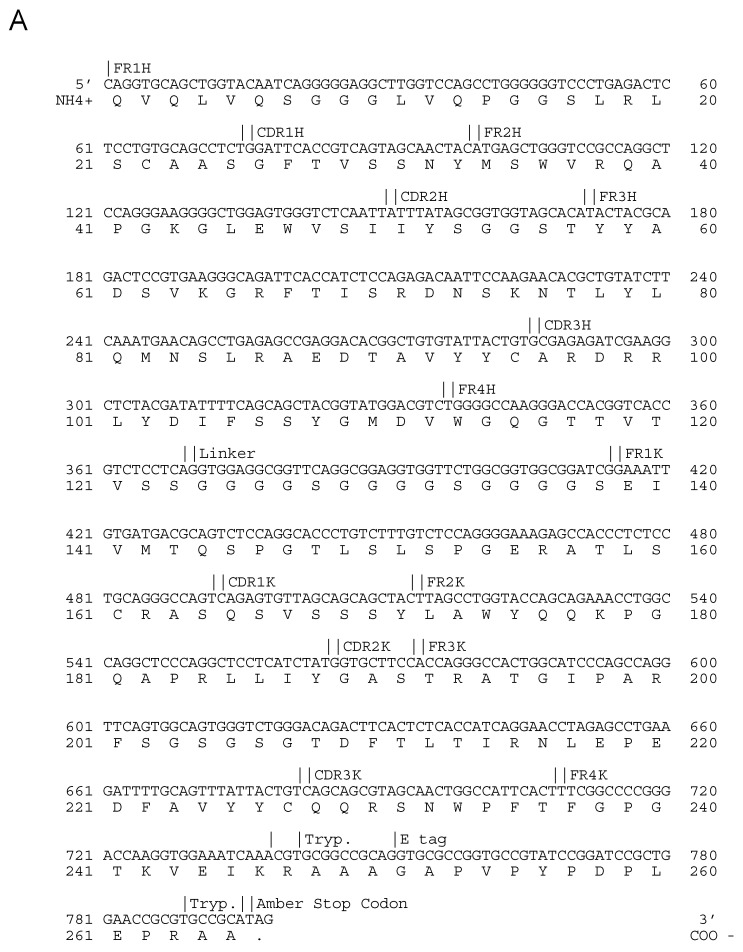

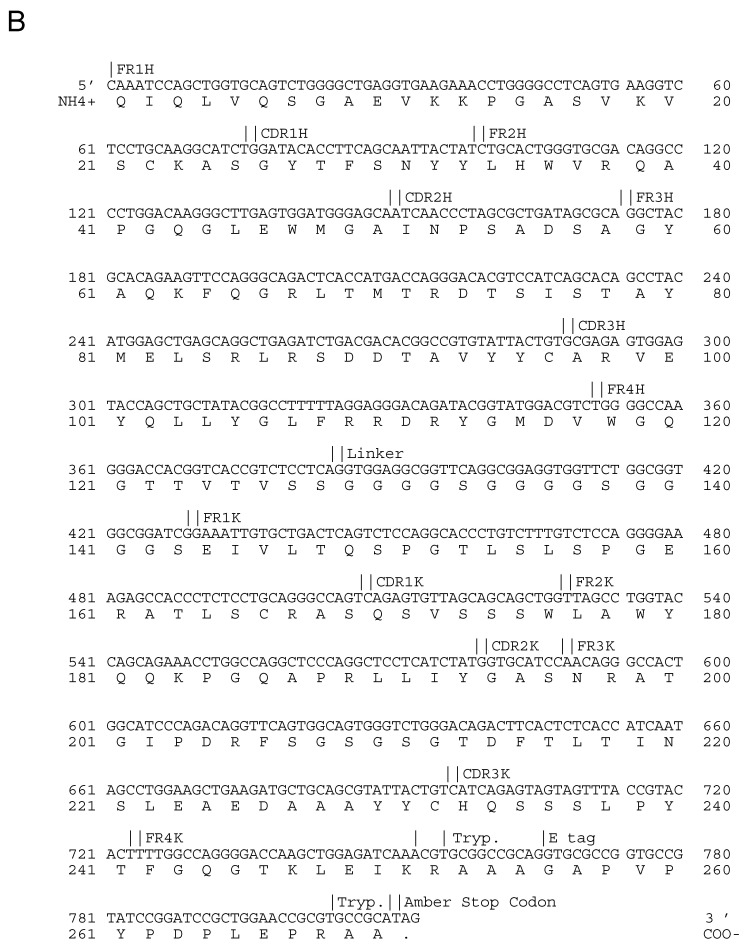
Deduced amino acid sequence of variable domains of heavy and light chains of the anti-VACV 1.2.2.H9 (**A**) and 1.4.1.C4 (**B**).

Specific antibodies were selected in four rounds of enrichment. After each round, 176 individual *E. coli* HB2151 colonies were isolated for the production of soluble antibodies in a microtiter plate format. Specific binding to VACV Elstree was tested in an ELISA. The background was determined on BSA. No specifically binding scFvs were observed after the first selection round. The second selection round revealed one clone with a 25 times higher absorbance over the background. The clone was designated as 1.2.2.H9. Two clones, both from the third and fourth round of selection, had absorbance at least four times over the background. In addition, the fourth round revealed one scFv, 1.4.1.C4, with an absorbance of around 33 times over the background. ScFv-1.2.2.H9 (Acc. No.: MW520863) and 1.4.1.C4 (Acc. No.: MW520864) were sequenced and, according to their deduced amino acid residues, classified to the human VH3/D3/JH6-VKIII/JK3 (1.2.2.H9) (Figure 3A) and VH1/D2/JH6-VKIII/JK2 (1.4.1.C4) V(D)J families (Figure 3B). 

Large-scale production and immune affinity purification were successful only for the scFv-1.2.2.H9, as the concentration of scFv-1.4.1.C4 was always very low. 

### 3.3. Binding Characteristics of scFv-1.2.2.H9, scFv-Fc-1.2.2.H9, and IgG1-1.2.2.H9 in ELISA

**Figure 4:**
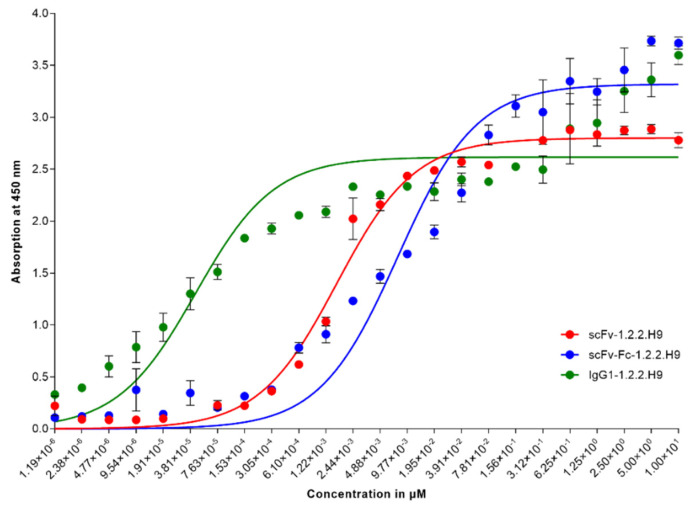
Indirect ELISAs with scFv-1.2.2.H9, scFv-Fc-1.2.2.H9, and IgG1-1.2.2. H9 on 2 µg/mL VACV Elstree in triplicates. The titration was performed in twofold serial dilutions. The starting concentrations were 10 µM of purified antibodies. The solid lines represent the interpolated curves used to calculate the binding kinetics according to Michaelis-Menten.

Correct insertion of the variable regions of scFv-1.2.2.H9 into the larger formats scFv-Fc-1.2.2.H9 and IgG1-1.2.2.H9 was confirmed by sequencing. ScFv-Fc fragments are bivalent, with an Fc part mediating effector functions. IgG1 represents a complete full-size antibody. Here, only the variable regions of scFv-1.2.2.H9 were cloned. Unlike in an scFv-Fc molecule, no linker fragment is required. The binding kinetics of all molecules were measured using an indirect ELISA with twofold serial dilutions in triplicate, starting with 10 µM of the respective antibody format (Figure 4), and calculated according to Michaelis-Menten [58]. 

The K_m_ of the scFv-1.2.2.H9 to VACV Elstree was 1.61 nM (0.045 μg/mL), with a corresponding v_max_ of 2.8 OD_450 nm_. The kinetic of the scFv-Fc-1.2.2.H9 was determined with 7.685 nM (0.402 μg/mL) and v_max_ = 3.319 OD_450 nm_. The lowest K_m_ of 43.8 pM (0.006 μg/mL) was calculated for the IgG1-1.2.2.H9 format. V_max_ was 2.617 OD_450 nm_.

**Figure 5:**
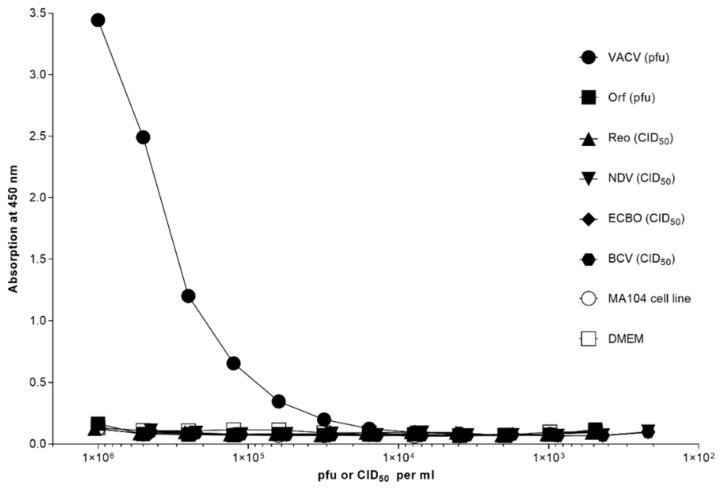
Capture ELISA with 25 µg/mL 1.2.2.H9 coated onto a 96-well microtiter plate. Different virus strains received from cell-cultured material were tested: 10^6^ pfu/mL vaccinia virus Elstree (VACV), 10^6^ pfu parapoxvirus orf D1701 (Orf), 10^6.75^ CID_50_/mL bovine coronavirus V270 (BCV), 10^7.625^ CID_50_/mL reovirus (Reo), 10^7.25^ CID_50_/mL Newcastle disease virus (NDV), and 10^7.25^ CID_50_/mL enterocytopathogenic bovine orphan virus (ECBO). Negative controls were MA104 cell line and DMEM. The titrations were conducted in twofold serial dilutions. Specific binding was detected using anti-MVA-polyclonal rabbit Ab (1:5000).

Distinct capture abilities were observed with the scFv-1.2.2.H9 for ≥1.5625 × 10^3^ pfu/mL VACV Elstree. No specific binding to scFv-1.2.2.H9 and the used detector anti-MVA-polyclonal rabbit Ab were seen with the other viruses tested (Figure 5).

### 3.4. Epitope Mapping

**Figure 6:**
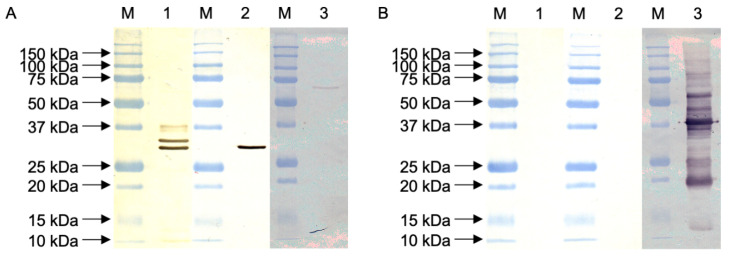
Western blot analysis for the detection of VACV Elstree ((**A**), blotted in lanes 1–3) and parapoxvirus orf D1701 ((**B**), blotted in lanes 1–3) as a negative control. Anti-MVA-polyclonal rabbit Ab/anti-rabbit pAb-HRP (1) served as a positive control for the detection of VACV Elstree. The specific detection of the 32 kDa protein D8 occurred with scFv-1.2.2.H9/anti-E tag mA-HRP (2) on the blotted VACV Elstree. Anti-orf polyclonal sheep Ab/anti-sheep pAb-HRP (3) was the positive control for orf D1701.

Western blot analysis revealed an epitope on the 32 k protein D8 of VACV Elstree (Figure 6A). No detection occurred on the parapoxvirus orf D1701 (Figure 6B). 

**Figure 7:**
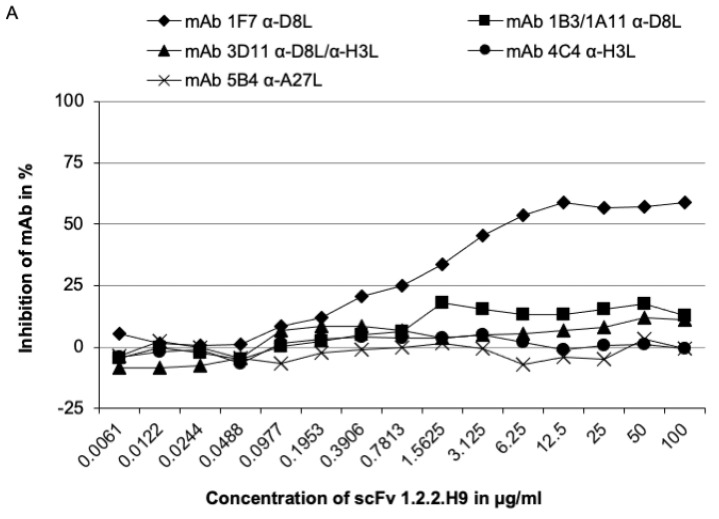

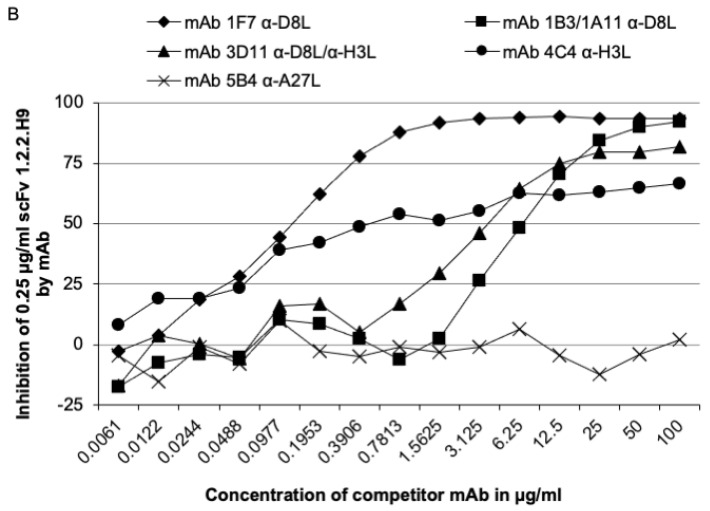
Inhibition of mAb in binding to their epitopes by scFv1.2.2.H9 with a starting concentration of 100 µg/mL scFv-1.2.2.H9 (**A**) and vice versa mAbs 1F7, 1B3/1A11, 3D11, 4C4, 5B4 (**B**). Microtiter plates were coated with 2 µg/mL VACV Elstree and the first scFv/mAbs were titrated. The results demonstrate the average from three independent assays. A reduction in the photometer adsorption (OD_450 nm_) of ≥50% indicated that two tested mAbs bind to identical or closely related antigenic sites. (**A**) Inhibition (≥50%) of mAb 1F7 was achieved with about 6.25 μg/mL scFv-1.2.2.H9. (**B**) The mAb 1F7 with at least 0.1953 µg/mL. More than 12.5 and 6.25 µg/mL of mAbs 1B3/1A11 and 3D11 is required to block the scFv-1.2.2.H9, respectively. A total of 0.7813 µg/mL of mAb 4C4 blocks the binding of scFv-1.2.2.H9.

The D8 protein harbors conformational epitopes. To define the amino acid residues of the 1.2.2.H9 epitope, typified monoclonal murine antibodies were used as competitors in an inhibition ELISA [35]. The scFv 1.2.2.H9 was able to block the mAb 1F7 up to 58%, whereas the scFv was blocked by the mAbs 1F7, 1B3/1A11, 3D11, and 4C4 in different concentrations (Figure 7). 

The mAb 1F7 inhibited ≥50% of the scFv-1.2.2.H9 with at least 0.1953 µg/mL and a maximum of 94%. A concentration of at least 0.7813 µg/mL mAb 4C4 was sufficient in the same assay. However, the curve did not exhibit a clear sigmoidal development. Here, we assume that the small molecule scFv-1.2.2.H9 can still pass to its epitope on D8. The highest concentrations of 6.25 µg/mL and 12.5 µg/mL, respectively, were needed to block the scFv-1.2.2.H9 by mAbs 3D11 and 1B3/1A11. The epitope of scFv-1.2.2.H9 can be assigned to 2B. 

### 3.5. In Vitro Neutralization

**Figure 8:**
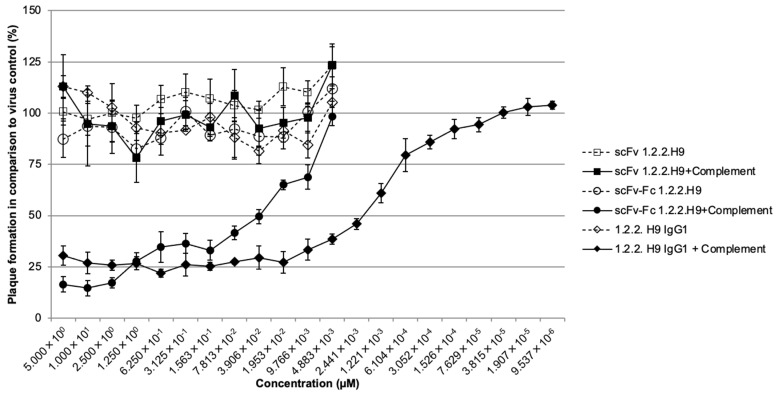
The PRNT was performed in triplicates with a starting concentration of 10 µM of the respective 1.2.2.H9 molecule. The scFv-1.2.2.H9 with (filled square) and without (unfilled square) complement, as well as the scFv-Fc-1.2.2.H9 and IgG1-1.2.2.H9 without (unfilled circle and diamond) human complement, showed no neutralization. Neutralization was observed after the addition of 1% human complement for both molecules with an Fc part (filled circle and diamond).

The classical plaque reduction neutralization test (PRNT) was performed in triplicates, with a starting concentration of 10 µM of the respective 1.2.2.H9 molecule. A reduction in plaque numbers of ≥50% was regarded as neutralization. The scFv-1.2.2.H9 with and without complement, as well as the scFv-Fc-1.2.2.H9 and IgG1-1.2.2.H9 without the addition of human complement, showed no neutralization. However, after the addition of 1% human complement, both molecules with an Fc part neutralized with 0.0776 µM and 0.01324 µM, respectively. Maximum neutralization was up to 75% for both molecules (Figure 8).

### 3.6. In Vivo Passive Protection

The in vivo passive protection of the three antibody formats against a VACV infection was investigated in NMRI mice. First, each of the three antibody formats and three control preparations were inoculated intraperitoneally into six mice per group. Negative control group mice were injected with either PBS or anti-EHV1 6B11 mAb (kindly provided by Hermann Meyer, Bundeswehr Institute of Microbiology, Munich, Germany). The positive control was anti-MVA-polyclonal rabbit Ab. Twenty-four hours after passive immunization, all mice were challenged with 4 LD50 VACV Munich 1. 

**Figure 9:**
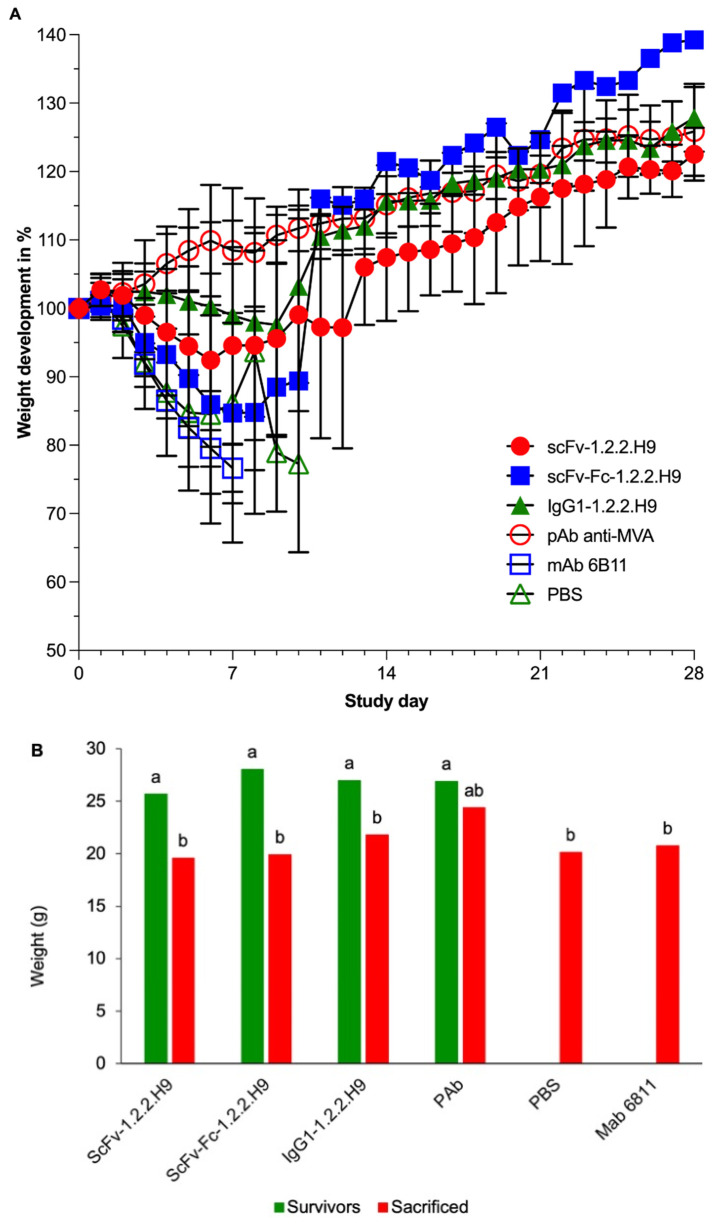
(**A**) Weight development and standard deviations per treatment group postvaccination on study day 0 and post-challenge on study day 1. The weight is plotted in proportion to the starting weight on study day 0. (**B**) Least square means for weight (in g) for the effect of survival ability of different treatment groups. Different letters illustrate the significant differences between the least square means of different factor levels (*p* < 0.05).

The weight development showed a drop in all groups, except group 4 (pAb anti-MVA), until about day 12 (group 1 scFv-1.2.2.H9) (Figure 9A). The Ls means of the weight of the surviving and sacrificed animals were significantly different in all groups, except for group 4 (Figure 9B). 

**Figure 10:**
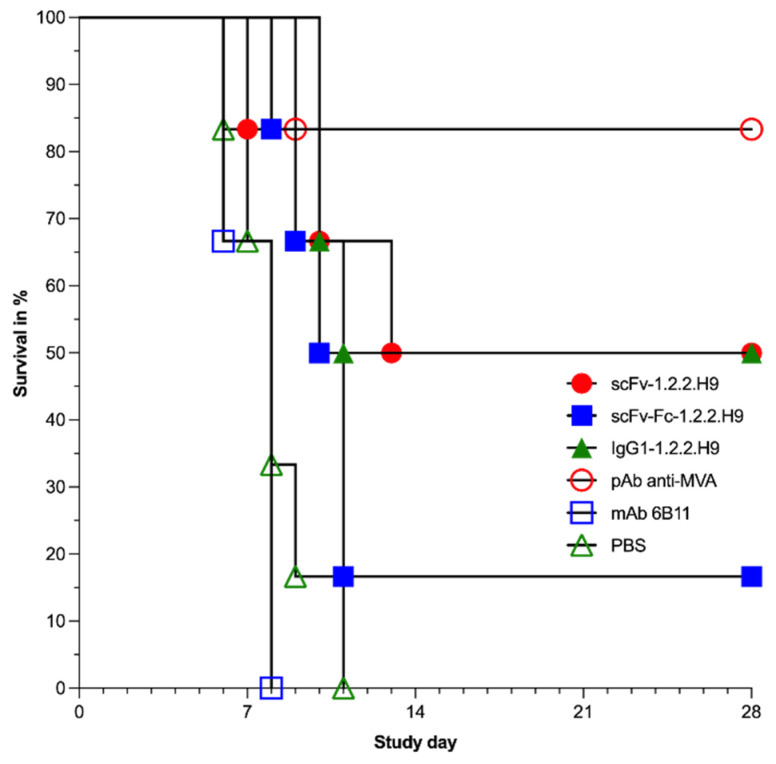
Survival rate in % of mice during the in vivo passive immunization.

**Figure 11:**
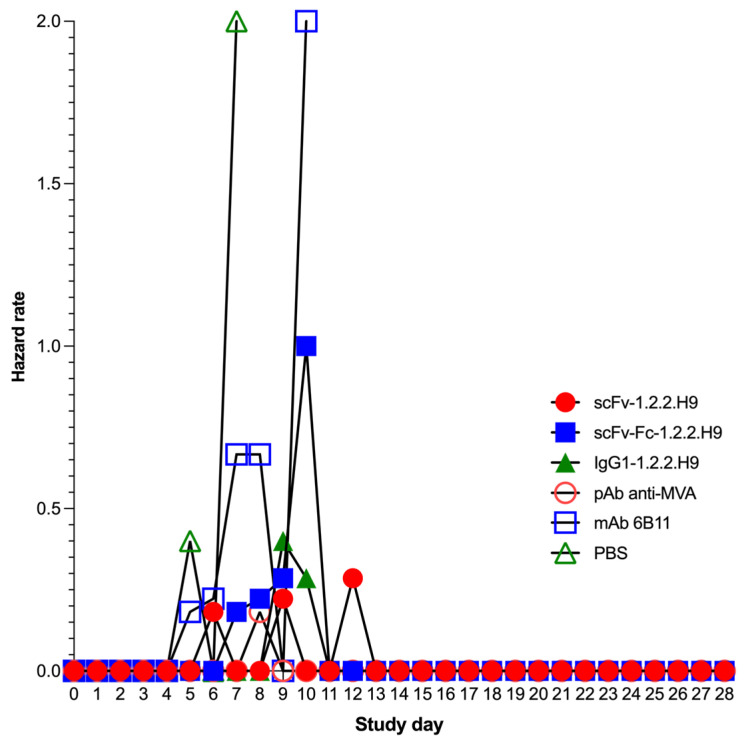
Hazard function for different treatment groups during the in vivo passive immunization.

Compared to the surviving animals, all of the sacrificed animals showed a significant decrease in weight development during the post-challenge time period, with the exception of the animals of the pAb anti-MVA group (group 4). This was characterized by the fact that this group exhibited an initial weight gain at the beginning of the experiment, followed by a significant decrease in weight development. This infection-related weight loss development was also reflected in the survival rate of the animals in the different treatment groups, as shown by the Kaplan-Meier survival analysis in Figure 10 and Figure 11. 

Of the mice receiving the scFv-1.2.2.H9 (group 1), three were protected and three were sacrificed at day 7, 10, and 11 post-challenge. Five out of six mice receiving scFv-Fc-1.2.2.H9 (group 2) were sacrificed between 8 and 11 days post-challenge. Three mice of the IgG1-1.2.2.H9 group (group 3) were sacrificed on days 10 and 11. One animal was sacrificed in the pAb anti-MVA positive control group (group 4) on day 9; the remaining animals survived the challenge until termination of the experiment on day 28. All animals of the two negative control groups (anti-EHV1 6B11 mAb and PBS, groups 5 and 6) were sacrificed between day 6 and 11. The higher survival rate resulted in a lower hazard rate at certain time points. The group treated with mAb 6B11 has a lower survival rate at a very early stage of infection, which is reflected in a high hazard rate in the period between 6 and 8 days post-infection. The same is more or less true for the PBS group. The lower survivability in the early phase post-infection also holds true for the animals in this treatment group, which also leads to a high hazard rate between 7 and 11 days post-infection. Likewise, the scFv-Fc-1.2.2.H9 treatment group exhibits lower survivability at these time windows, and thus a higher hazard rate. Compared to the previous two groups, the scFv-1.2.2.H9 treatment group has an extended time window for mortality with a lower intensity, so that hazard rates are manifested intermittently from 7 to 13 days after infection. The onset of later mortality in the IgG1-1.2.2.H9 group leads to a later onset of hazard in this group. The low mortality rate on day 10 post-infection in this group leads to a low hazard rate.

Comparison of the survival curves revealed a significant effect on group 1 to group 6 (*p* = 0.0087). The survival curve of group 2 was significantly different (*p* = 0.0387) to group 4. In addition, group 4 was significantly different to group 5 (*p* = 0.0167) and group 6 (*p* = 0.0003). Survival of group 3 was significantly different (*p* = 0.0069) to group 6. Table 2 summarizes the statistical findings for the survival. The threshold of ≥50% survival was only achieved by treatments with scFv-1.2.2.H9 and IgG1-1.2.2.H9, as well as the positive control pAb αMVA.

**Figure 12:**
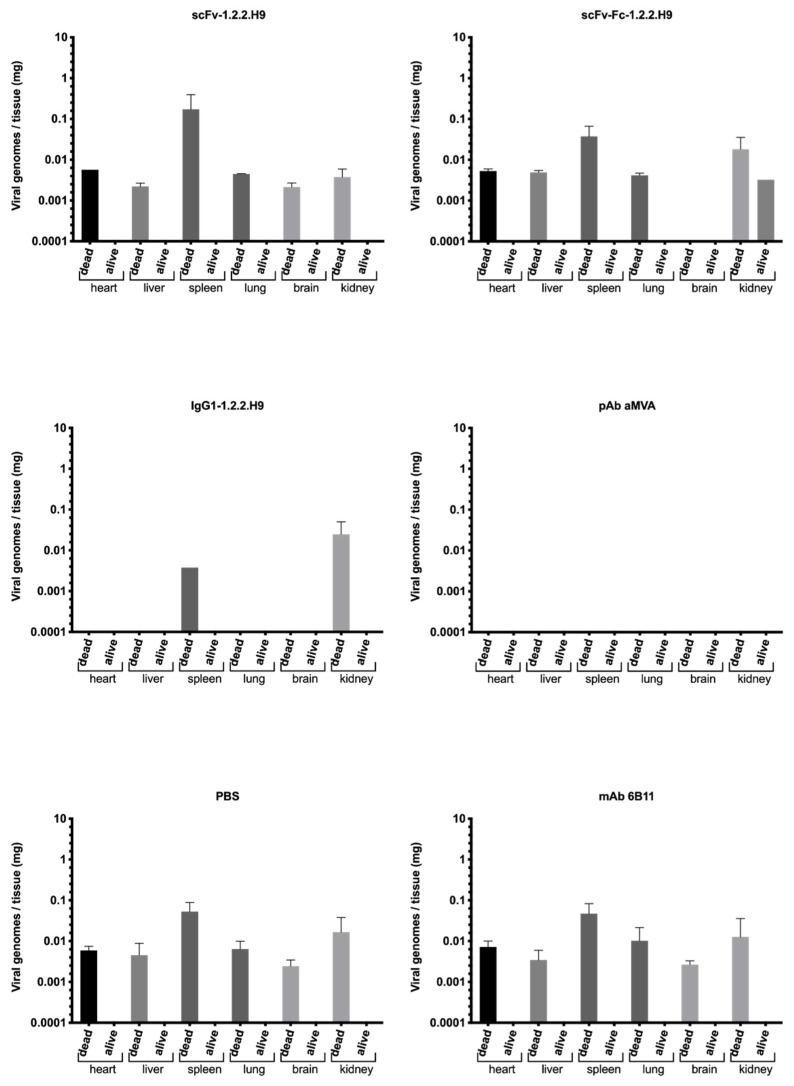
Concentration of the VACV DNA in different organs of mice after the challenge of VACV Munich 1. The mean of the viral load of sacrificed vs. surviving mice is shown for each treatment group.

At the end of the experiment (day 28 post-challenge), heart, liver, spleen, lung, brain, and kidney of all animals were harvested and examined for viral loads by real-time PCR (Figure 12). 

The highest viral load of the scFv-1.2.2.H9-inoculated mice (group 1) was found in the spleen, followed by the heart, lungs, and kidneys, while lower viral loads were identified in the brain and liver on average. The spleen of surviving mice also showed a high viral load until day 28, whereas the viral load dropped but was still detectable in other organs of surviving mice. The scFv-Fc-1.2.2.H9-inoculated mice (group 2) showed a higher viral load in the spleen and kidney, followed by heart, liver, and lung, while the IgG1-1.2.2.H9-inoculated mice (group 3) showed viral loads only in the kidney and spleen. Group 2 surviving animals also had detectable viral replication in the kidney on day 28. No virus was found in the organs of the mice inoculated with the pAb anti-MVA (group 4). One deceased animal also did not drop significantly in weight and might have died due to reasons other than the poxvirus infection. In the case of anti-EHV1 mAb 6B11 (group 5) and PBS (group 6), the highest viral load was found in the spleen, followed by the kidney, lung, heart, liver, and brain. The PBS group had the highest titers in all organs.

**Figure 13:**
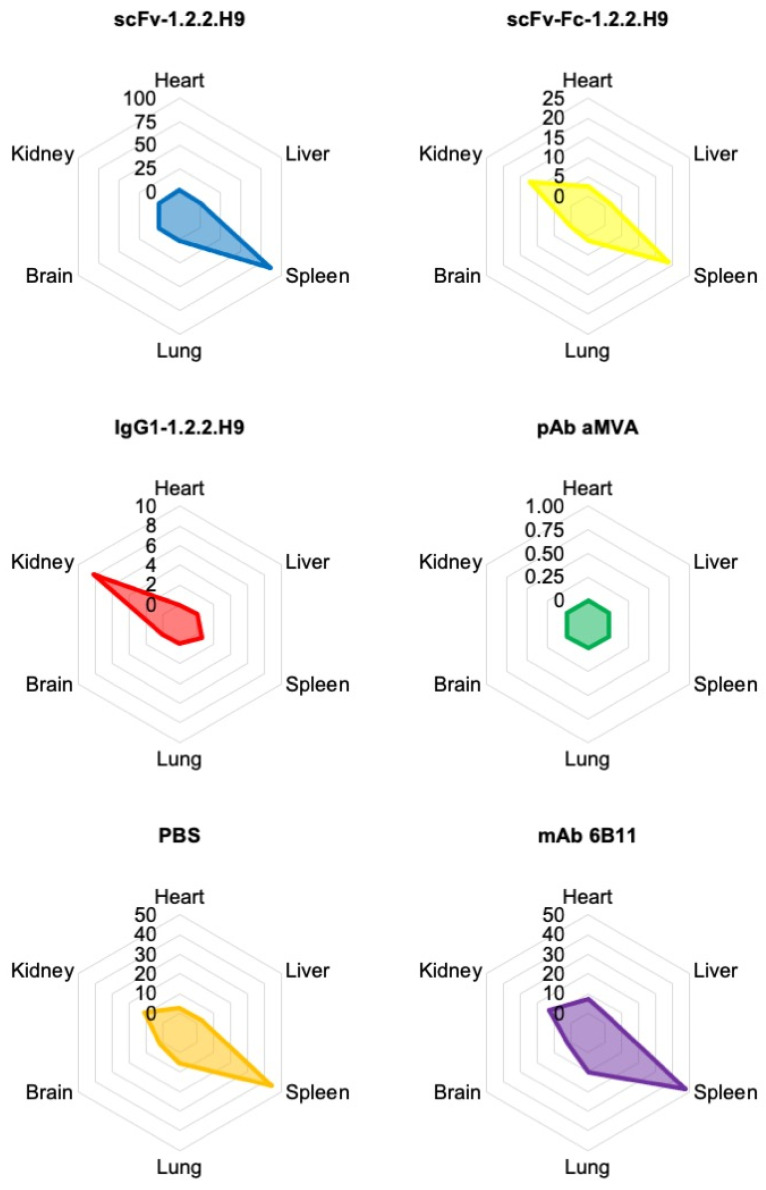
The radar plots show the mean viral loads of the respective treatment groups in genome copies/g tissue.

The mean viral load in genome copies per g tissue of the different treatments including all animals is shown in a radar chart (Figure 13).

The highest virus load was observed in the spleen for treatment with scFv-1.2.2.H9, followed by treatments with mAb 6B11, PBS, and scFv-Fc-1.2.2.H9, while no or low viral load was observed for treatments with pAb anti-MVA and IgG1-1.2.2.H9. Overall, the spleen appears to be the organ most affected with viral load across all treatments, except for the group which received IgG1-1.2.2.H9. In this case, the kidney was primarily affected. The viral load in the different organs is partly reflected in the survival probability. Treatment with PBS, mAb 6B11, and scFv-Fc-1.2.2.H9 leads to a significant decrease in survival probability, while treatment with pAb anti-MVA, where no viral load was detected, shows the highest survival probability. The relatively high viral load in the scFv-1.2.2.H9 group is associated to an intermediate survival probability.

## 4. Discussion

For the development of OPXV-specific recombinant human scFv antibodies, the IgG repertoire of four donors vaccinated intracutaneously with live vaccinia virus (VACV) vaccine was amplified, cloned, and displayed onto M13K07 phage. In this study, the target of the scFv-1.2.2.H9 was assigned to the 32 kDa D8 protein of VACV, which is part of the MV, the most common infectious form [61]. The function of the conserved OPXV D8 protein is the adsorption of virus to the host cell surface due to its binding to chondroitin sulfate [22,38]. The reciprocal blocking effect of scFv-1.2.2.H9 and the mAb 1F7/2F9 led to the conclusion that the target of scFv-1.2.2.H9 is the same or part of the epitope #2B recognized by mAb 1F7/2F9 [35]. Moreover, this study demonstrates the partial neutralizing activity against VACV using only one monoclonal scFv, as the smallest antibody-derived molecule. The scFv-1.2.2.H9 was sufficient to protect 50% of mice from a lethal challenge with VACV Munich 1. Comparison of the hazard rates of all groups shows that the time window between 6 and 13 days post-infection is a critical phase for survival of the different groups. The negative controls, PBS and mAb 6B11, have very high hazard rates very early post-infection due to early and high mortality in these groups. Viral genome copies were still detected on day 28 in surviving mice, suggesting either a decreased or delayed replication in survivors. Several studies confirmed the feasibility of protection against orthopoxvirus infections using monoclonal antibodies targeting neutralizing epitopes of the EEV and IMV [49,62,63,64]. The obtained scFv-1.2.2.H9 was engineered to a full-size antibody to improve the binding affinity and effector function. The IgG1-1.2.2.H9 was also sufficient to protect 50% of mice after challenging with VACV Munich 1, whereas the scFv-Fc-1.2.2.H9 was only able to protect 16.7%. Steric effects or secondary modifications of the molecule leading to different pharmacokinetics might explain this phenomenon, because the viral load seemed to decrease with increasing molecule sizes. The difference of the in vivo results to the in vitro complement-dependent neutralization can also not be explained by a lack of complement binding. The residues Glu 318, Lys 320, and Lys 322, which are responsible for complement binding, are relatively conserved in other antibody isotypes, such as all human IgGs and in mouse IgG2a, IgG2b, and IgG3 [65]. Moreover, the Fc part of the scFv-Fc is the same as for the IgG1 molecule. The protection of mice in vivo was, in general, associated with a reduction in viral loads in liver, lung, kidney, heart, spleen, and brain compared to the negative control with PBS. The scFv, scFv-Fc, as well as the IgG1-1.2.2.H9 reduced viral dissemination to internal organs; however, they did not fully protect mice from death. The reduction in the viral loads to the organs depended on the size of the antibody. In contrast to the scFv-1.2.2.H9, which also reduced the dissemination to some degree, the IgG1-1.2.2.-H9 almost completely inhibited the spread. This is related to the effector functions mentioned above and a putative longer half-life. In in vitro studies, the VACV-neutralizing abilities of the scFv-Fc-1.2.2.H9 and the IgG1-1.2.2.H9 were improved by the addition of 1% human complement, while the addition of complement had no effect on the scFv-1.2.2.H9 because of the lacking Fc region. Other authors characterized anti-D8 mAbs with VACV-neutralizing abilities only in the presence of complement, because complement is needed to increase the footprint of the mAbs [36]. These complement-dependent findings were also confirmed for mAbs directed to A27, A33, D8, H3, L1 [31,32], and B5 [63,66]. Schmaljohn et al. [46] were the first to establish a recombinant human Fab library and select specific binding molecules to a number of VACV proteins. Two Fabs have bound to a 35 kDa protein, while one Fab was precipitated with a 34 kDa protein. Those Fabs were able to neutralize in vitro. Three additional neutralizing Fabs were observed after the addition of anti-Fab antibodies. While our library with higher diversity was constructed from peripheral B-lymphocytes of four different volunteers, others selected B5-specific recombinant Fabs from an immunized chimpanzee library [33], where B-cells were isolated from bone marrow 11 weeks after immunization. A majority of 80–90% of antigen-specific plasma cells resides from day 45 in the bone marrow and can be detected for at least one year after immunization [67]. To avoid an invasive intervention to the study volunteers, we preferred to collect blood samples and isolate peripheral blood lymphocytes. There, the amount of specific B-cells can be determined as about 20% [46]. Therefore, we expect a high amount of VACV unspecific antibodies within the library, which can be disadvantageous in the selection procedure. Western blotting analysis of the unselected hyperphage pool revealed the possibility of selecting scFv against other VACV proteins, e.g., using protein directed panning methods. The goal in passive and therapeutic protection might be the combination of EV- and MV-specific monoclonal antibodies, which can block a poxvirus infection at different stages. This is supported by work dissecting the neutralizing immune response in human sera from vaccinated donors [28,29]. Well-characterized monoclonal antibodies produced in GMP-controlled cell culture systems or plants, for example, harbor the advantage of maintaining the same quality and functionality. Moreover, data on antigenic sites for cross-reacting or monospecific neutralizing antibodies are of high relevance for target-directed screening of human immunoglobulin libraries to generate specifically engineered human recombinant antibodies, which might help in controlling any future outbreak of zoonotic orthopoxviruses. 

## 5. Conclusions

The study demonstrated the selection of specific binding fragments out of an immunized human scFv library generated from peripheral B-lymphocytes. The further characterized scFv-1.2.2.H9 was converted into larger formats by adding an Fc part to permit effector functions. In vitro neutralization required binding of the complement. The D8 protein was identified as the target, and the epitope was mapped by competition ELISA using a panel of mAbs. ScFv and IgG1-1.2.2.H9 showed partial protection properties in an in vivo mouse model.

## Figures and Tables

**Table 1:** Monoclonal antibodies (mAb) [35] used in an inhibition ELISA for the identification of the target of scFv 1.2.2.H9.

Epitope ID	MAb	Isotype	Virus Strain Used for mAb Production	Isotype
2A	1B3/1A11	IgG2a	VACV M1	IgG2a
2B	1F7/2F9	IgG2b	ECTV M1	IgG2b
2D	3D11/2G7	IgG2a	CPXV KR2 Brighton	IgG1
2G	4C4/2B6	IgG2a	CPXV KR2 Brighton	IgG1

**Table 2:** Statistical comparison of the survival curves using a log-rank test. Group 1 was immunized with scFv-1.2.2.H9, group 2 received scFv-Fc-1.2.2.H9. Group 3 was immunized with IgG1-1.2.2.H9. The positive group 4 was treated with pAb anti-MVA. The negative controls, group 5 and 6, received anti-EHV1 6B11 mAb and PBS, respectively.

Equality Test over Levels				
Test		Chi-Quadrat	DF	Pr > Chi-Quadrat
Log-Rank		20.8477	5	0.0009
Adjustment for Multiple Comparisons for the Log-Rank Test				
Strata Comparison		Chi-Quadrat	*p*-Values	
Group	Group		Raw	Sidak Adjustment
1	2	1.2919	0.2557	0.9881
1	3	0.00968	0.9216	1.0000
1	4	0.8085	0.3686	0.9990
1	5	2.1580	0.1418	0.8992
1	6	6.8927	0.0087	0.1222
2	3	1.4918	0.2219	0.9768
2	4	4.2725	0.0387	0.4471
2	5	0.1233	0.7255	1.0000
2	6	1.9182	0.1661	0.9344
3	4	0.6326	0.4264	0.9998
3	5	2.3846	0.1225	0.8593
3	6	7.3013	0.0069	0.0985
4	5	5.7236	0.0167	0.2237
4	6	13.4020	0.0003	0.0038
5	6	0.9339	0.3339	0.9977

## Data Availability

Raw data is available upon request.

## Supplementary Materials

The following are available online at https://www.mdpi.com/article/10.3390/vaccines9111308/s1. **Table S1:** Degenerate primer used for the amplification of variable- and part of the constant region of the heavy IgG-chains and κ- and λ-light chains respectively. The expected product size was 650 bp. **Table S2:** Degenerate primer used for the amplification of variable regions with overlapping parts coding for the (G_4_S)_3_-linker. The expected product sizes were 420 bp for the variable region of the heavy chains and 380 bp for the light chains. The BACK-primers used for the PCR of the heavy chain variable region were the same shown in Supplemental Table S1. **Figure S1:** Amplification of the 650 bp fragments of the IgG heavy chains from the cDNA synthesized from four different B-lymphocyte donors (A to D). The fragments consisted of the variable and parts of the constant regions. Primers amplifying GAPDH (11) were chosen to monitor the quality of the cDNA. S1 and S2 were the 1 kb and 100 bp ladders respectively. The products occurred from the combination of HuIgG1-4CH1FOR with HuVH1aBACK (1), HuVH1bBACK (2), HuVH1cBACK (3), HuVH1d-7BACK (4), HuVH2aBACK (5), HuVH3aBACK (6), HuVH4aBACK (7), HuVH5aBACK (8), HuVH6aBACK (9), and HuVH7aBACK (10). The no-template control as negative control is applied in lane 12. **Figure S2:** Amplification of the 650 bp fragments of the κ light chains from the cDNA synthesized from four different B-lymphocyte donors (A to D). The fragments consisted of the variable and parts of the constant regions. Primers amplifying GAPDH (11) were chosen to monitor the quality of the cDNA. S1 and S2 were the 1 kb and 100 bp ladders respectively. The products occurred from the combination of HuCκFOR with HuVκ1aBACK (1), HuVκ1bBACK (2), HuVκ2aBACK (3), HuVκ2bBACK (4), HuVκ3aBACK (5), HuVκ3cBACK (6), HuVκ4aBACK (7), HuVκ5aBACK (8), HuVκ6aBACK (9), and HuVκ6aBACK (10). The no-template control as negative control is applied in lane 12. **Figure S3:** Amplification of the 650 bp fragments of the λ light chains from the cDNA synthesized from four different B-lymphocyte donors (A to D). The fragments consisted of the variable and parts of the constant regions. Primers amplifying GAPDH (12) were chosen to monitor the quality of the cDNA. S1 and S2 were the 1 kb and 100 bp ladders respectively. The products occurred from the combination of HuCλ2FOR and HuCλ7FOR in equal amounts with HuVλ1aBACK (1), HuVλ1bBACK (2), HuVλ2BACK (3), HuVλ3aBACK (4), HuVλ3bBACK (5), HuVλ4a-9BACK (6), HuVλ4bBACK (7), HuVλ5BACK (8), HuVλ6BACK (9), HuVλ7-8BACK (10), and HuVλ10BACK (11). The no-template control as negative control is applied in lane 13. **Figure S4:** Amplification of the variable regions of the heavy chains (A), κ light chains (B), and λ light chains (C) from pooled 650 bp samples (1–10, and 11 for the λ variable regions). The fragments consisted of the variable region and overlapping parts coding for the (G_4_S)_3_-linker. S1 and S2 were the 1 kb and 100 bp ladders respectively. The no-template control as negative control is applied in lane 11, and 12 for the λ variable regions respectively. The products were gel-purified and taken as template for splicing by overlap extension-PCR. **Figure S5:** Amplification of full scFv including restriction sites for *Sfi*I and *Not*I. The pooled variable regions of the heavy chains were either connected with the variable regions of κ light chains (A), or λ light chains (B). After SOE-PCR (splicing by overlap extension) the products were divided into 10 templates (1–10) for the re-amplification and connection of restriction sites. S1 and S2 were the 1 kb and 100 bp ladders respectively. The no-template control as negative control is applied in lane 11. The products were gel-purified and restricted before ligation into pCANTAB5E. **Figure S6:** (A) Original Western blots as presented in Figure 2 and (B) Original Western blots as presented in Figure 6 in the main manuscript. After development blot strips were dried, cut to form, and scanned.

## References

[1] Moss B. (2006). Poxvirus entry and membrane fusion. Virology.

[2] Fenner F., Henderson D.A., Arita I., Jezek Z., Ladnyi I.D. (1988). Smallpox and Its Eradication.

[3] Ladnyi I.D., Breman J.G. (1978). Smallpox eradication: Progress and problems. Dev. Biol. Stand..

[4] Kurth A., Wibbelt G., Gerber H.P., Petschaelis A., Pauli G., Nitsche A. (2008). Rat-to-elephant-to-human transmission of cowpox virus. Emerg. Infect. Dis..

[5] Becker C., Kurth A., Hessler F., Kramp H., Gokel M., Hoffmann R., Kuczka A., Nitsche A. (2009). Cowpox virus infection in pet rat owners: Not always immediately recognized. Dtsch. Arztebl. Int..

[6] Vorou R.M., Papavassiliou V.G., Pierroutsakos I.N. (2008). Cowpox virus infection: An emerging health threat. Curr. Opin. Infect. Dis..

[7] Campe H., Zimmermann P., Glos K., Bayer M., Bergemann H., Dreweck C., Graf P., Weber B.K., Meyer H., Büttner M. (2009). Cowpox virus transmission from pet rats to humans, Germany. Emerg. Infect. Dis..

[8] Ladnyj I.D., Ziegler P., Kima E. (1972). A human infection caused by monkeypox virus in Basankusu Territory, Democratic Republic of the Congo. Bull. World Health Organ..

[9] Reed K.D., Melski J.W., Graham M.B., Regnery R.L., Sotir M.J., Wegner M.V., Kazmierczak J.J., Stratman E.J., Li Y., Fairley J.A. (2004). The detection of monkeypox in humans in the Western Hemisphere. N. Engl. J. Med..

[10] Vaughan A., Aarons E., Astbury J., Balasegaram S., Beadsworth M., Beck C.R., Chand M., O’Connor C., Dunning J., Ghebrehewet S. (2018). Two cases of monkeypox imported to the United Kingdom, September 2018. Eurosurveill.

[11] Fulginiti V.A. (2003). Risks of smallpox vaccination. JAMA.

[12] Cono J., Casey C.G., Bell D.M. (2003). Smallpox vaccination and adverse reactions. Guidance for clinicians. MMWR Recomm. Rep..

[13] Fulginiti V.A., Papier A., Lane J.M., Neff J.M., Henderson D.A. (2003). Smallpox vaccination: A review, part II. Adverse events. Clin. Infect. Dis..

[14] Hopkins R.J., Lane J.M. (2004). Clinical efficacy of intramuscular vaccinia immune globulin: A literature review. Clin. Infect. Dis..

[15] Hopkins R.J., Kramer W.G., Blackwelder W.C., Ashtekar M., Hague L., Winker-La Roche S.D., Berezuk G., Smith D., Leese P.T. (2004). Safety and pharmacokinetic evaluation of intravenous vaccinia immune globulin in healthy volunteers. Clin. Infect. Dis..

[16] Kempe C.H. (1960). Studies smallpox and complications of smallpox vaccination. Pediatrics.

[17] Feery B.J. (1976). The efficacy of vaccinial immune globulin. A 15-year study. Vox Sang..

[18] Sawyer L.A. (2000). Antibodies for the prevention and treatment of viral diseases. Antivir. Res..

[19] Smith G.L., Vanderplasschen A., Law M. (2002). The formation and function of extracellular enveloped vaccinia virus. J. Gen. Virol..

[20] Boulter E.A., Appleyard G. (1973). Differences between extracellular and intracellular forms of poxvirus and their implications. Prog. Med. Virol..

[21] Blasco R., Moss B. (1992). Role of cell-associated enveloped vaccinia virus in cell-to-cell spread. J. Virol..

[22] Hsiao J.C., Chung C.S., Chang W. (1999). Vaccinia virus envelope D8L protein binds to cell surface chondroitin sulfate and mediates the adsorption of intracellular mature virions to cells. J. Virol..

[23] Lin C.L., Chung C.S., Heine H.G., Chang W. (2000). Vaccinia virus envelope H3L protein binds to cell surface heparan sulfate and is important for intracellular mature virion morphogenesis and virus infection in vitro and in vivo. J. Virol..

[24] Rodriguez J.F., Janeczko R., Esteban M. (1985). Isolation and characterization of neutralizing monoclonal antibodies to vaccinia virus. J. Virol..

[25] Wallengren K., Risco C., Krijnse-Locker J., Esteban M., Rodriguez D. (2001). The A17L gene product of vaccinia virus is exposed on the surface of IMV. Virology.

[26] Wolffe E.J., Vijaya S., Moss B. (1995). A myristylated membrane protein encoded by the vaccinia virus L1R open reading frame is the target of potent neutralizing monoclonal antibodies. Virology.

[27] Ichihashi Y., Oie M. (1996). Neutralizing epitope on penetration protein of vaccinia virus. Virology.

[28] Benhnia M.R., McCausland M.M., Su H.P., Singh K., Hoffmann J., Davies D.H., Felgner P.L., Head S., Sette A., Garboczi D.N. (2008). Redundancy and plasticity of neutralizing antibody responses are cornerstone attributes of the human immune response to the smallpox vaccine. J. Virol..

[29] Eto A., Fujita M., Nishiyama Y., Saito T., Molina D.M., Morikawa S., Saijo M., Shinmura Y., Kanatani Y. (2019). Profiling of the antibody response to attenuated LC16m8 smallpox vaccine using protein array analysis. Vaccine.

[30] Xu C., Meng X., Yan B., Crotty S., Deng J., Xiang Y. (2011). An epitope conserved in orthopoxvirus A13 envelope protein is the target of neutralizing and protective antibodies. Virology.

[31] Ahsendorf H.P., Gan L.L., Eltom K.H., Abd El Wahed A., Hotop S.K., Roper R.L., Beutling U., Broenstrup M., Stahl-Hennig C., Hoelzle L.E. (2019). Species-Specific Conservation of Linear Antigenic Sites on Vaccinia Virus A27 Protein Homologs of Orthopoxviruses. Viruses.

[32] Kaever T., Matho M.H., Meng X., Crickard L., Schlossman A., Xiang Y., Crotty S., Peters B., Zajonc D.M. (2016). Linear Epitopes in Vaccinia Virus A27 Are Targets of Protective Antibodies Induced by Vaccination against Smallpox. J. Virol..

[33] Chen Z., Earl P., Americo J., Damon I., Smith S.K., Zhou Y.H., Yu F., Sebrell A., Emerson S., Cohen G. (2006). Chimpanzee/human mAbs to vaccinia virus B5 protein neutralize vaccinia and smallpox viruses and protect mice against vaccinia virus. Proc. Natl. Acad. Sci. USA.

[34] Aldaz-Carroll L., Whitbeck J.C., Ponce de Leon M., Lou H., Hirao L., Isaacs S.N., Moss B., Eisenberg R.J., Cohen G.H. (2005). Epitope-mapping studies define two major neutralization sites on the vaccinia virus extracellular enveloped virus glycoprotein B5R. J. Virol..

[35] Czerny C.P., Johann S., Holzle L., Meyer H. (1994). Epitope detection in the envelope of intracellular naked orthopox viruses and identification of encoding genes. Virology.

[36] Matho M.H., Maybeno M., Benhnia M.R., Becker D., Meng X., Xiang Y., Crotty S., Peters B., Zajonc D.M. (2012). Structural and biochemical characterization of the vaccinia virus envelope protein D8 and its recognition by the antibody LA5. J. Virol..

[37] Gilchuk I., Gilchuk P., Sapparapu G., Lampley R., Singh V., Kose N., Blum D.L., Hughes L.J., Satheshkumar P.S., Townsend M.B. (2016). Cross-Neutralizing and Protective Human Antibody Specificities to Poxvirus Infections. Cell.

[38] Maa J.S., Rodriguez J.F., Esteban M. (1990). Structural and functional characterization of a cell surface binding protein of vaccinia virus. J. Biol. Chem..

[39] Matho M.H., de Val N., Miller G.M., Brown J., Schlossman A., Meng X., Crotty S., Peters B., Xiang Y., Hsieh-Wilson L.C. (2014). Murine anti-vaccinia virus D8 antibodies target different epitopes and differ in their ability to block D8 binding to CS-E. PLoS Pathog..

[40] Mirzakhanyan Y., Gershon P. (2019). The Vaccinia virion: Filling the gap between atomic and ultrastructure. PLoS Pathog..

[41] Rodriguez J.R., Rodriguez D., Esteban M. (1992). Insertional inactivation of the vaccinia virus 32-kilodalton gene is associated with attenuation in mice and reduction of viral gene expression in polarized epithelial cells. J. Virol..

[42] Niles E.G., Seto J. (1988). Vaccinia virus gene D8 encodes a virion transmembrane protein. J. Virol..

[43] Sakhatskyy P., Wang S., Chou T.H., Lu S. (2006). Immunogenicity and protection efficacy of monovalent and polyvalent poxvirus vaccines that include the D8 antigen. Virology.

[44] Matho M.H., Schlossman A., Gilchuk I.M., Miller G., Mikulski Z., Hupfer M., Wang J., Bitra A., Meng X., Xiang Y. (2018). Structure-function characterization of three human antibodies targeting the vaccinia virus adhesion molecule D8. J. Biol. Chem..

[45] Hoogenboom H.R., de Bruine A.P., Hufton S.E., Hoet R.M., Arends J.W., Roovers R.C. (1998). Antibody phage display technology and its applications. Immunotechnology.

[46] Schmaljohn C., Cui Y., Kerby S., Pennock D., Spik K. (1999). Production and characterization of human monoclonal antibody Fab fragments to vaccinia virus from a phage-display combinatorial library. Virology.

[47] Frey A., Di Canzio J., Zurakowski D. (1998). A statistically defined endpoint titer determination method for immunoassays. J. Immunol. Methods.

[48] Hurteau G.J., Spivack S.D. (2002). mRNA-specific reverse transcription-polymerase chain reaction from human tissue extracts. Anal. Biochem..

[49] Czerny C.P., Mahnel H. (1990). Structural and functional analysis of orthopoxvirus epitopes with neutralizing monoclonal antibodies. J. Gen. Virol..

[50] Lowry O.H., Rosebrough N.J., Farr A.L., Randall R.J. (1951). Protein measurement with the Folin phenol reagent. J. Biol. Chem..

[51] Czerny C.P., Waldmann R., Scheubeck T. (1997). Identification of three distinct antigenic sites in parapoxviruses. Arch. Virol..

[52] Brochet X., Lefranc M.P., Giudicelli V. (2008). IMGT/V-QUEST: The highly customized and integrated system for IG and TR standardized V-J and V-D-J sequence analysis. Nucleic Acids Res..

[53] Giudicelli V., Brochet X., Lefranc M.P. (2011). IMGT/V-QUEST: IMGT standardized analysis of the immunoglobulin (IG) and T cell receptor (TR) nucleotide sequences. Cold Spring Harb. Protoc..

[54] Steinwand M., Droste P., Frenzel A., Hust M., Dubel S., Schirrmann T. (2013). The influence of antibody fragment format on phage display based affinity maturation of IgG. mAbs.

[55] Durocher Y., Perret S., Kamen A. (2002). High-level and high-throughput recombinant protein production by transient transfection of suspension-growing human 293-EBNA1 cells. Nucleic Acids Res..

[56] Jager V., Bussow K., Wagner A., Weber S., Hust M., Frenzel A., Schirrmann T. (2013). High level transient production of recombinant antibodies and antibody fusion proteins in HEK293 cells. BMC Biotech..

[57] Laemmli U.K. (1970). Cleavage of structural proteins during the assembly of the head of bacteriophage T4. Nature.

[58] Michaelis L., Menten M.L. (1913). Die Kinetik der Invertinwirkung. Biochem. Z..

[59] Czerny C.P., Mahnel H., Hornstein O. (1989). Testing the immunity to orthopoxviruses in the white mouse with vaccinia virus. Zent. Vet. B.

[60] Czerny C.-P., Alex M., Pricelius J., Zeller-Lue C., Meuer S., Wittwer C. (2001). Development of quantitative PCR tests for the detection of the orthopox virus adsorption protein gene (ORF D8L) on the Light CyclerTM. Rapid Real Time PCR—A Light Cycler Application.

[61] Smith G.L., Vanderplasschen A. (1998). Extracellular enveloped vaccinia virus. Entry, egress, and evasion. Adv. Exp. Med. Biol..

[62] McCausland M.M., Benhnia M.R., Crickard L., Laudenslager J., Granger S.W., Tahara T., Kubo R., Koriazova L., Kato S., Crotty S. (2010). Combination therapy of vaccinia virus infection with human anti-H3 and anti-B5 monoclonal antibodies in a small animal model. Antivir. Ther..

[63] Benhnia M.R., McCausland M.M., Laudenslager J., Granger S.W., Rickert S., Koriazova L., Tahara T., Kubo R.T., Kato S., Crotty S. (2009). Heavily isotype-dependent protective activities of human antibodies against vaccinia virus extracellular virion antigen B5. J. Virol..

[64] Ramirez J.C., Tapia E., Esteban M. (2002). Administration to mice of a monoclonal antibody that neutralizes the intracellular mature virus form of vaccinia virus limits virus replication efficiently under prophylactic and therapeutic conditions. J. Gen. Virol..

[65] Duncan A.R., Winter G. (1988). The binding site for C1q on IgG. Nature.

[66] Benhnia M.R., McCausland M.M., Moyron J., Laudenslager J., Granger S., Rickert S., Koriazova L., Kubo R., Kato S., Crotty S. (2009). Vaccinia virus extracellular enveloped virion neutralization in vitro and protection in vivo depend on complement. J. Virol..

[67] Slifka M.K., Matloubian M., Ahmed R. (1995). Bone marrow is a major site of long-term antibody production after acute viral infection. J. Virol..

