# Supplementary material for: Characterization of an In Vivo Neutralizing Anti-Vaccinia Virus D8 Single-Chain Fragment Variable (scFv) from a Human Anti-Vaccinia Virus-Specific Recombinant Library
Source: Vaccines (Basel). 2021 Nov 10;9(11):1308. doi: 10.3390/vaccines9111308 (PMC8619513; doi:10.3390/vaccines9111308)
Supplement: Supplementary file 1 [file vaccines-09-01308-s001.zip › vaccines-1422790-supplementary.pdf]

Legends to supplemental material

Table S1. Degenerate primer used for the amplification of variable- and part of the constant region of the heavy IgG-chains and κ- and λ-light chains respectively. The expected product size was 650 bp.

Primer	Sequence 5'→3'
HuIgG1-4CH1FOR	GTC CAC CTT GGT GTT GCT GGG CTT
HuVH1aBACK	CAR RTG CAG CTG GTG CAG TCT GG
HuVH1bBACK	CAG GTY CAG CTK GTG CAG TCT GG
HuVH1cBACK	SAG GTC CAG CTG GTA CAG TCT GG
HuVH1d-7BACK	CAR RTS CAG CTG GTG CAR TCT GG
HuVH2aBACK	CAG RTC ACC TTG ARG GAG TCT GG
HuVH3aBACK	SAG GTR CAG CTG GTG GAG TCT GG
HuVH4aBACK	CAG STG CAG CTG CAG GAG TCS GG
HuVH5aBACK	GAR GTG CAG CTG GTG CAG TCT GG
HuVH6aBACK	CAG GTA CAG CTG CAG CAG TCA GG
HuVH7aBACK	CAG GTS CAG CTG GTG CAA TCW GG
HuCκFOR	AGA CTC TCC CCT GTT GAA GCT CTT
HuVκ1aBACK	RAC ATC CAG WTG ACC CAG TCT CC
HuVκ1bBACK	GMC ATC CAG TTG ACC CAG TCT CC
HuVκ2aBACK	GAT RTT GTG ATG ACY CAG WCT CC
HuVκ2bBACK	GAT RTT GTG ATG ACW CAG TCT CC
HuVκ3aBACK	GAA ATW GTG WTG ACR CAG TCT CC
HuVκ3cBACK	GAA ATT GTR WTG ACA CAG TCT CC
HuVκ4aBACK	GAC ATC GTG ATG ACC CAG TCT CC
HuVκ5aBACK	GAA ACG ACA CTC ACG CAG TCT CC
HuVκ6aBACK	GAA ATT GTG CTG ACT CAG TCT CC
HuVκ6bBACK	GAT GTT GTG ATG ACA CAG TCT CC
HuCλ2FOR	TGA AGA TTC TGT AGG GGC CAC TGT CTT
HuCλ7FOR	AGA GCA TTC TGC AGG GGC CAC TGT CTT
HuVλ1aBACK	CAG TCT GTG CTG ACT CAG CCA CC
HuVλ1bBACK	CAG TCT GTG YTG ACG CAG CCG CC
HuVλ2BACK	CAG TCT GCC CTG ACT CAG CCT GC
HuVλ3aBACK	TCC TAT GWG CTG ACW CAG CCA CC
HuVλ3bBACK	TCT TCT GAG CTG ACT CAG GAC CC
HuVλ4a-9BACK	CWG CCT GTG CTG ACT CAG CCM CC
HuVλ4bBACK	CAG CYT GTG CTG ACT CAA TCR
HuVλ5BACK	CAG SCT GTG CTG ACT CAG CCR
HuVλ6BACK	AAT TTT ATG CTG ACT CAG CCC CA
HuVλ7-8BACK	CAG RCT GTG GTG ACY CAG GAG CC
HuVλ10BACK	CAG GCA GGG CTG ACT CAG CCA CC

Table S2. Degenerate primer used for the amplification of variable regions with overlapping parts coding for the $(G_4S)_3$-linker. The expected product sizes were 420 bp for the variable region of the heavy chains and 380 bp for the light chains. The BACK-primers used for the PCR of the heavy chain variable region were the same shown in Supplemental Table S1.

Primer	Sequence 5'→3'
Linker-HuJH1-2	AGA ACC ACC TCC GCC TGA ACC GCC TCC ACC TGA GGA GAC GGT GAC CAG GGT GC
Linker-HuJH3	AGA ACC ACC TCC GCC TGA ACC GCC TCC ACC TGA AGA GAC GGT GAC CAT TGT CC
Linker-HuJH4-5	AGA ACC ACC TCC GCC TGA ACC GCC TCC ACC TGA GGA GAC GGT GAC CAG GGT TC-
Linker-HuJH6	AGA ACC ACC TCC GCC TGA ACC GCC TCC ACC TGA GGA GAC GGT GAC CGT GGT CC
HuJκ1FOR	ACG TTT GAT TTC CAC CTT GGT CCC
HuJκ2FOR	ACG TTT GAT CTC CAG CTT GGT CCC
HuJκ3FOR	ACG TTT GAT ATC CAC TTT GGT CCC
HuJκ4FOR	ACG TTT GAT CTC CAC CTT GGT CCC
HuJκ5FOR	ACG TTT AAT CTC CAG TCG TGT CCC
Linker-HuVκ1a	GTT CAG GCG GAG GTG GTT CTG GCG GTG GCG GAT CGR ACA TCC AGW TGA CCC AGT CTC C
Linker-HuVκ1b	GTT CAG GCG GAG GTG GTT CTG GCG GTG GCG GAT CGG MCA TCC AGT TGA CCC AGT CTC C
Linker-HuVκ2a	GTT CAG GCG GAG GTG GTT CTG GCG GTG GCG GAT CGG ATR TTG TGA TGA CYC AGW CTC C
Linker-HuVκ2b	GTT CAG GCG GAG GTG GTT CTG GCG GTG GCG GAT CGG ATR TTG TGA TGA CWC AGT CTC C
Linker-HuVκ3a	GTT CAG GCG GAG GTG GTT CTG GCG GTG GCG GAT CGG AAA TWG TGW TGA CRC AGT CTC C
Linker-HuVκ3c	GTT CAG GCG GAG GTG GTT CTG GCG GTG GCG GAT CGG AAA TTG TRW TGA CAC AGT CTC C
Linker-HuVκ4a	GTT CAG GCG GAG GTG GTT CTG GCG GTG GCG GAT CGG ACA TCG TGA TGA CCC AGT CTC C
Linker-HuVκ5a	GTT CAG GCG GAG GTG GTT CTG GCG GTG GCG GAT CGG AAA CGA CAC TCA CGC AGT CTC C
Linker-HuVκk6a	GTT CAG GCG GAG GTG GTT CTG GCG GTG GCG GAT CGG AAA TTG TGC TGA CTC AGT CTC C
Linker-HuVκ6b	GTT CAG GCG GAG GTG GTT CTG GCG GTG GCG GAT CGG ATG TTG TGA TGA CAC AGT CTC C
Hu Jλ1FOR	ACC TAG GAC GGT GAC CTT GGT CCC

Hu Jλ2-3FOR	ACC TAG GAC GGT CAG CTT GGT CCC
Hu Jλ4-5FOR	ACC TAA AAC GGT GAG CTG GGT CCC
Hu Jλ7FOR	ACC GAG GAC GGT CAG CTG GGT GCC
Linker-HuVλ1a	GTT CAG GCG GAG GTG GTT CTG GCG GTG GCG GAT CGC AGT CTG TGC TGA CTC AGC CAC C
Linker-HuVλ1b	GTT CAG GCG GAG GTG GTT CTG GCG GTG GCG GAT CGC AGT CTG TGY TGA CGC AGC CGG C
Linker-HuVλ2	GTT CAG GCG GAG GTG GTT CTG GCG GTG GCG GAT CGC AGT CTG CCC TGA CTC AGC CTG C
Linker-HuVλ3a	GTT CAG GCG GAG GTG GTT CTG GCG GTG GCG GAT CGT CCT ATG WGC TGA CWC AGC CAC C-
Linker-HuVλ3b	GTT CAG GCG GAG GTG GTT CTG GCG GTG GCG GAT CGT CTT CTG AGC TGA CTC AGG ACC C
Linker-HuVλ4a-9	GTT CAG GCG GAG GTG GTT CTG GCG GTG GCG GAT CGC WGC CTG TGC TGA CTC AGC CMC C
Linker-HuVλ4b	GTT CAG GCG GAG GTG GTT CTG GCG GTG GCG GAT CGC AGC YTG TGC TGA CTC AAT CRG C
Linker-HuVλ5	GTT CAG GCG GAG GTG GTT CTG GCG GTG GCG GAT CGC AGS CTG TGC TGA CTC AGC CRT C
Linker-HuVλ6	GTT CAG GCG GAG GTG GTT CTG GCG GTG GCG GAT CGA ATT TTA TGC TGA CTC AGC CCC A
Linker-HuVλ7-8	GTT CAG GCG GAG GTG GTT CTG GCG GTG GCG GAT CGC AGR CTG TGG TGA CYC TGG AGC C
Linker-HuVλ10	GTT CAG GCG GAG GTG GTT CTG GCG GTG GCG GAT CGC AGG CAG GGC TGA CTC AGC CAC C

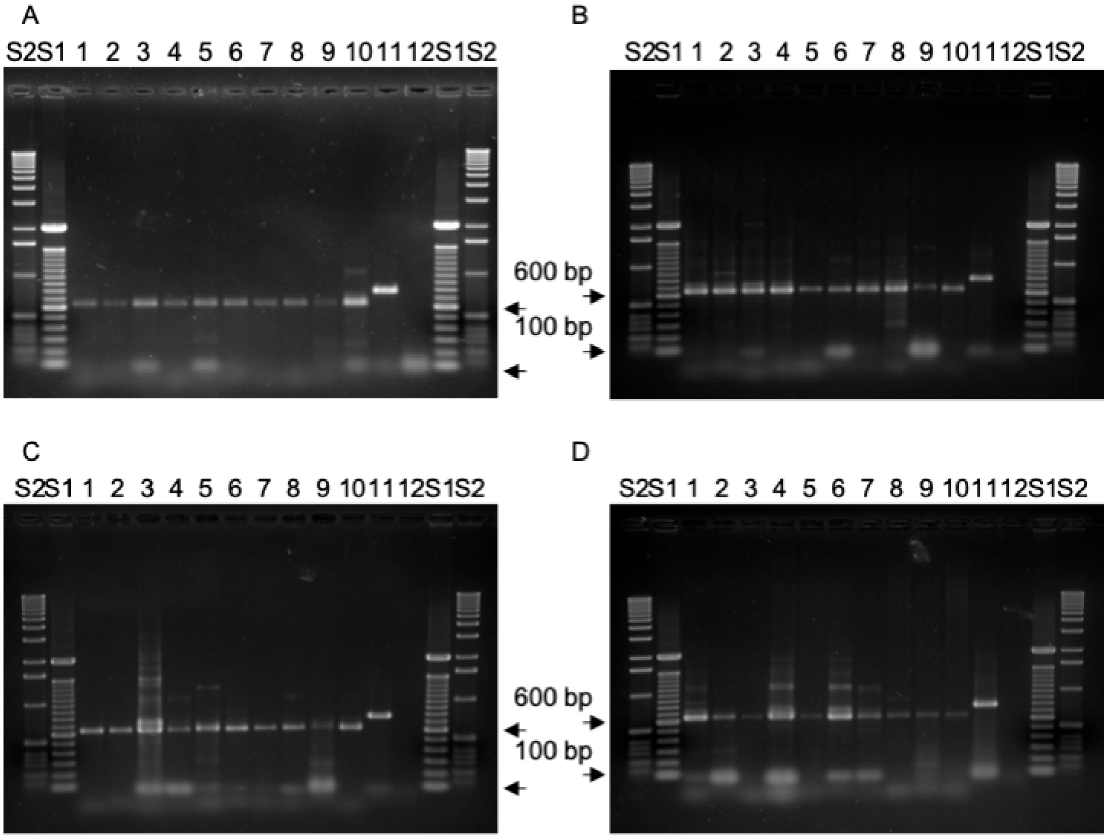

Figure S1. Amplification of the 650 bp fragments of the IgG heavy chains from the cDNA synthesized from four different B-lymphocyte donors (**A** to **D**). The fragments consisted of the variable and parts of the constant regions. Primers amplifying GAPDH (11) were chosen to monitor the quality of the cDNA. S1 and S2 were the 1 kb and 100 bp ladders respectively. The products occurred from the combination of HuIgG1-4CH1FOR with HuVH1aBACK (1), HuVH1bBACK (2), HuVH1cBACK (3), HuVH1d-7BACK (4), HuVH2aBACK (5), HuVH3aBACK (6), HuVH4aBACK (7), HuVH5aBACK (8), HuVH6aBACK (9), and HuVH7aBACK (10). The no-template control as negative control is applied in lane 12.

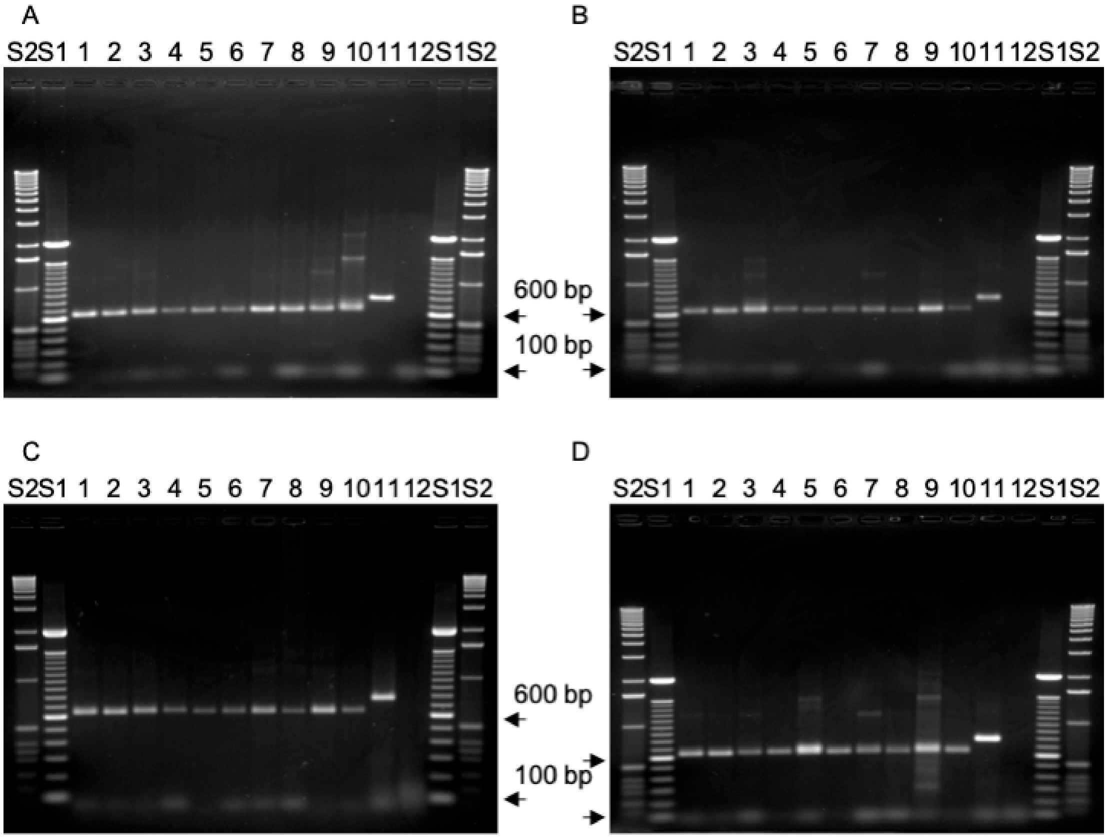

Figure S2. Amplification of the 650 bp fragments of the κ light chains from the cDNA synthesized from four different B-lymphocyte donors (**A** to **D**). The fragments consisted of the variable and parts of the constant regions. Primers amplifying GAPDH (11) were chosen to monitor the quality of the cDNA. S1 and S2 were the 1 kb and 100 bp ladders respectively. The products occurred from the combination of HuCκFOR with HuVκ1aBACK (1), HuVκ1bBACK (2), HuVκ2aBACK (3), HuVκ2bBACK (4), HuVκ3aBACK (5), HuVκ3cBACK (6), HuVκ4aBACK (7), HuVκ5aBACK (8), HuVκ6aBACK (9), and HuVκ6aBACK (10). The no-template control as negative control is applied in lane 12.

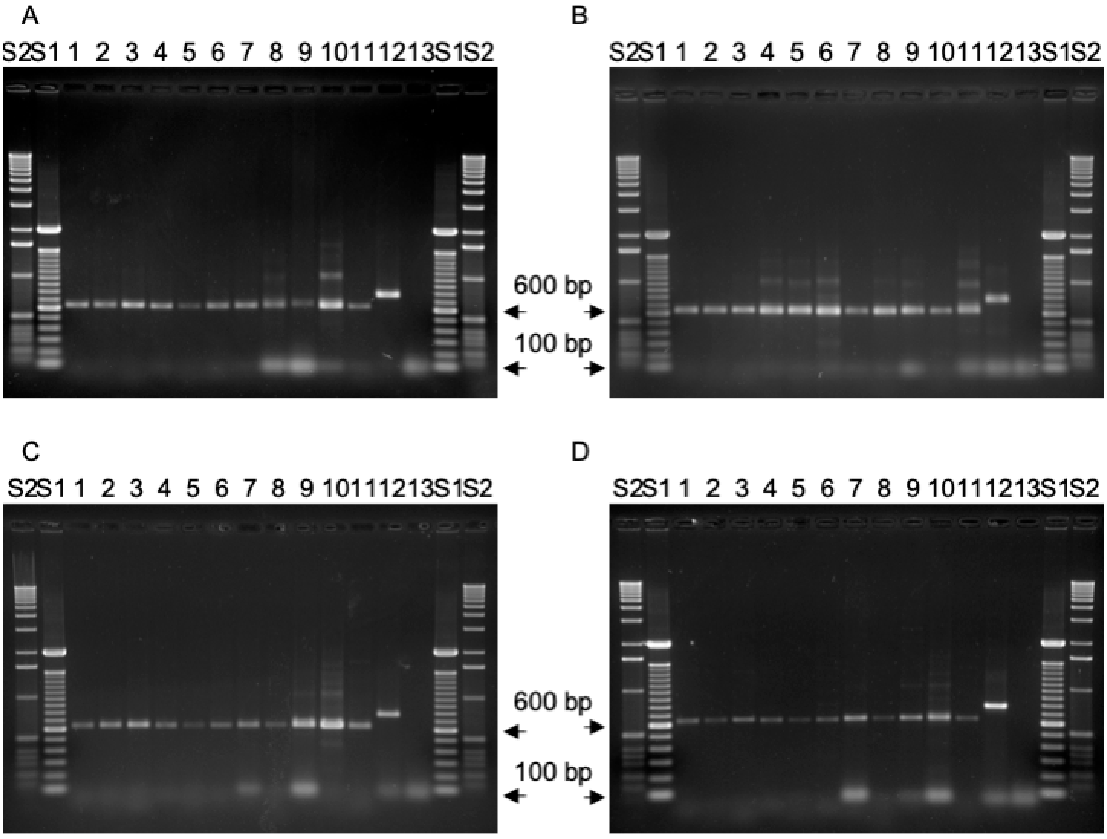

Figure S3. Amplification of the 650 bp fragments of the λ light chains from the cDNA synthesized from four different B-lymphocyte donors (**A** to **D**). The fragments consisted of the variable and parts of the constant regions. Primers amplifying GAPDH (12) were chosen to monitor the quality of the cDNA. S1 and S2 were the 1 kb and 100 bp ladders respectively. The products occurred from the combination of HuCλ2FOR and HuCλ7FOR in equal amounts with HuVλ1aBACK (1), HuVλ1bBACK (2), HuVλ2BACK (3), HuVλ3aBACK (4), HuVλ3bBACK (5), HuVλ4a-9BACK (6), HuVλ4bBACK (7), HuVλ5BACK (8), HuVλ6BACK (9), HuVλ7-8BACK (10), and HuVλ10BACK (11). The no-template control as negative control is applied in lane 13.

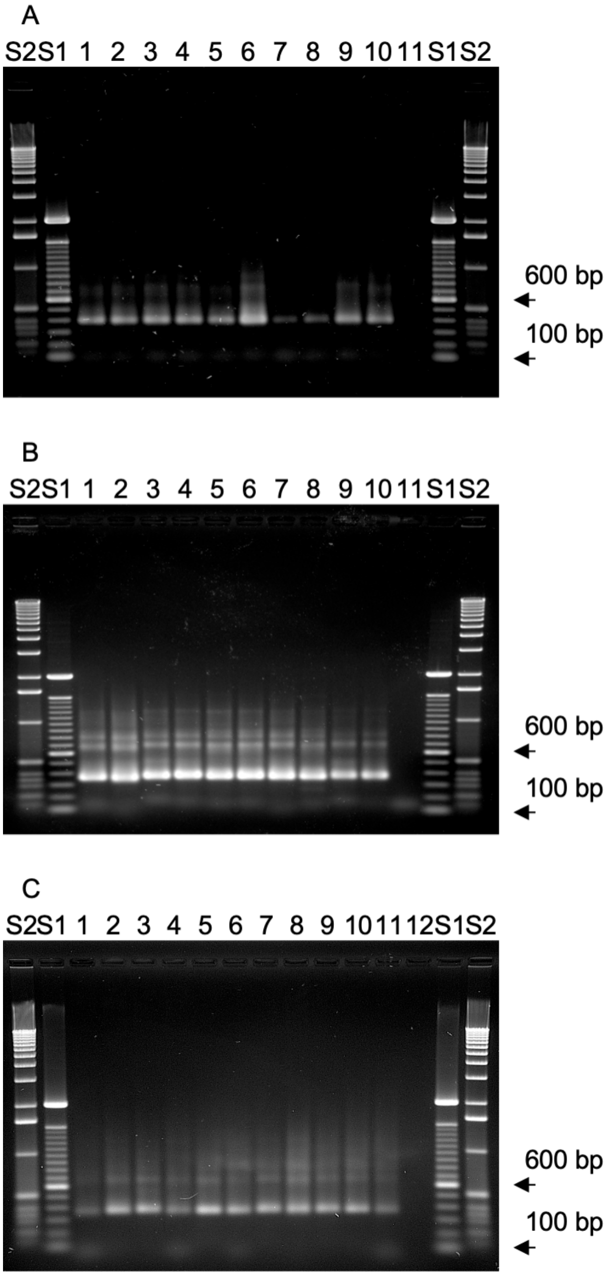

Figure S4. Amplification of the variable regions of the heavy chains (**A**), κ light chains (**B**), and λ light chains (**C**) from pooled 650 bp samples (1–10, and 11 for the λ variable regions). The fragments consisted of the variable region and overlapping parts coding for the $(G_4S)_3$-linker. S1 and S2 were the 1 kb and 100 bp ladders respectively. The no-template control as negative control is applied in lane 11, and 12 for the λ variable regions respectively. The products were gel-purified and taken as template for splicing by overlap extension-PCR.

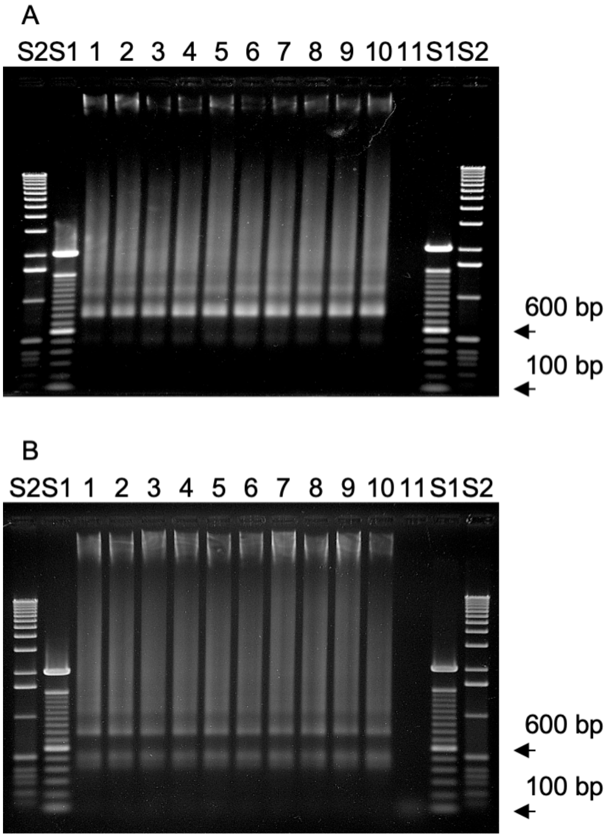

Figure S5. Amplification of full scFv including restriction sites for *Sfi*I and *Not*I. The pooled variable regions of the heavy chains were either connected with the variable regions of κ light chains (**A**), or λ light chains (**B**). After SOE-PCR (splicing by overlap extension) the products were divided into 10 templates (1–10) for the re-amplification and connection of restriction sites. S1 and S2 were the 1 kb and 100 bp ladders respectively. The no-template control as negative control is applied in lane 11. The products were gel-purified and restricted before ligation into pCANTAB5E.

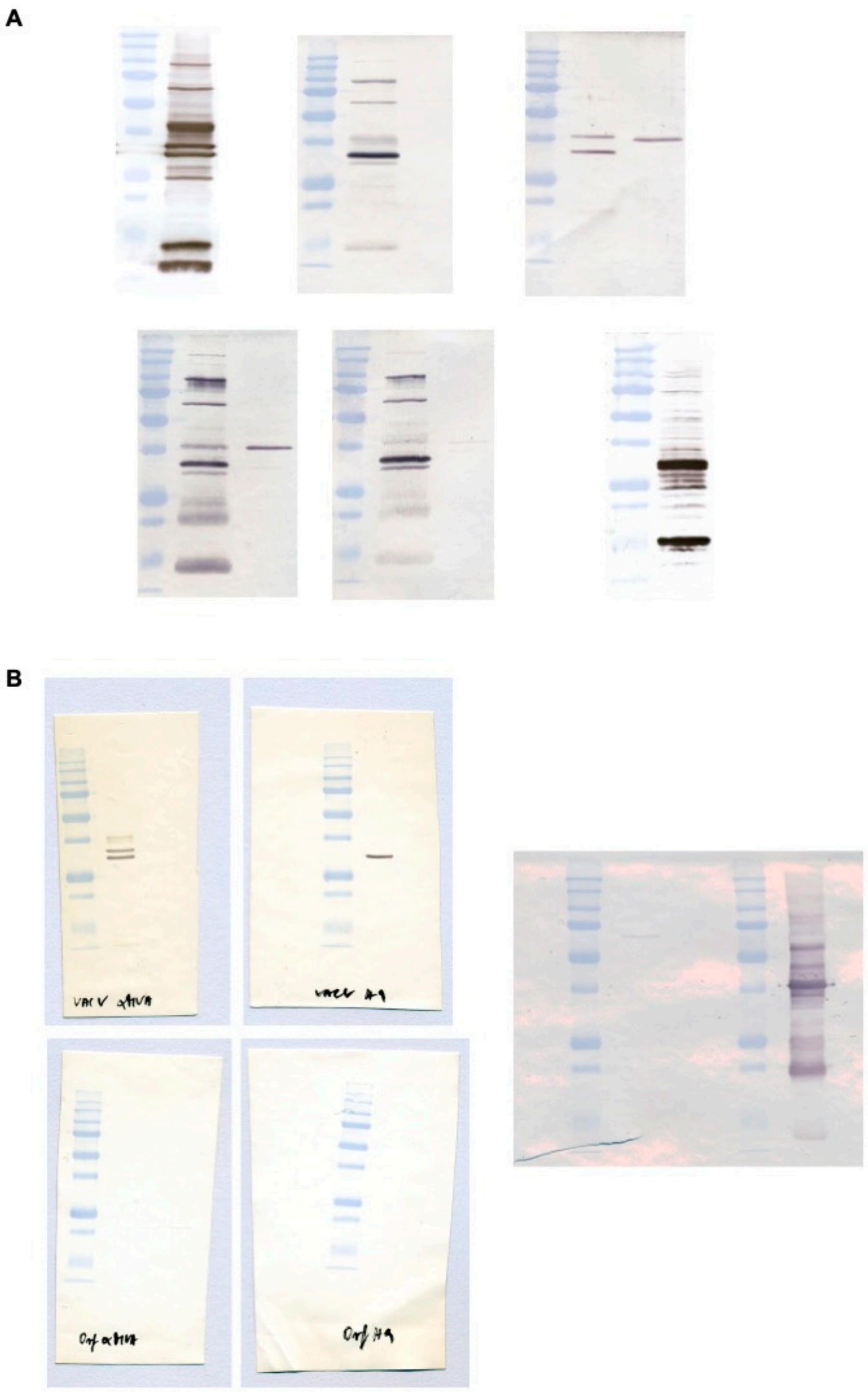

Figure S6. (**A**) Original Western blots as presented in Figure 2 and (**B**) Original Western blots as presented in Figure 6 in the main manuscript. After development blot strips were dried, cut to form, and scanned.
